# Bayesian time-aligned factor analysis of paired multivariate time series

**Published:** 2021

**Authors:** Arkaprava Roy, Jana Schaich Borg, David B Dunson

**Affiliations:** Department of Biostatistics, University of Florida, Gainnesville, FL 32611, USA; Social Science Research Institute, Duke University, Durham, NC 27708-0251, USA; Department of Statistical Science, Duke University, Durham, NC 27708-0251, USA

**Keywords:** CIFA, Dynamic factor model, Hamiltonian Monte Carlo, JIVE, Monotonicity, Paired time series, Social mimicry, Time alignment, Warping

## Abstract

Many modern data sets require inference methods that can estimate the shared and individual-specific components of variability in collections of matrices that change over time. Promising methods have been developed to analyze these types of data in static cases, but only a few approaches are available for dynamic settings. To address this gap, we consider novel models and inference methods for pairs of matrices in which the columns correspond to multivariate observations at different time points. In order to characterize common and individual features, we propose a Bayesian dynamic factor modeling framework called Time Aligned Common and Individual Factor Analysis (TACIFA) that includes uncertainty in time alignment through an unknown warping function. We provide theoretical support for the proposed model, showing identifiability and posterior concentration. The structure enables efficient computation through a Hamiltonian Monte Carlo (HMC) algorithm. We show excellent performance in simulations, and illustrate the method through application to a social mimicry experiment.

## 1. Introduction

Many fields are routinely collecting matrix-variate data and asking questions about the similarity between subsets of those data. As the collection of these types of data expands, so does the need for new statistical methods that can capture the shared and individual-specific structure in multiple matrices, especially when matrices in a collection consist of multivariate observations collected over time. Here, we are motivated by the particular challenge of measuring the coordination between two people interacting dynamically. Many scientific questions require measurements of how similar the movements and expressions of two people are in these cases, because such similarity has been shown to be related to many interested phenomena and behaviors, including much people like each other or cooperate (Lakin and Chartrand, 2003; Johnston, 2002; Marsh et al., 2016). To address these questions, videos of social interactions are typically recorded, and the coordinates of different facial and body features from each individual in the pair are extracted over time. The data for each individual form a matrix, with the columns corresponding to different time points. One component of the variability in the two matrices will be attributable to shared structure, such as the patterns in which lips tend to move during conversation. Another component will be attributable to variability specific to each individual, such as differences in smile shapes, camera placements, sitting postures, and head sizes. When people interact, they often subconsciously imitate each other, but who initiates the imitation and the speed at which the imitation occurs varies over time. Thus, modeling the similarity in these paired dynamic matrix-variate data requires a strategy that can accommodate: 1) complex multivariate dependence among variables, and 2) dynamic time-varying lags between the two multivariate time series. Although our motivating example is from human social interactions, similar challenges are posed by other types of paired multivariate data, such as that collected in animal behavior studies, cellular imaging studies, finance, or handwriting recognition where there is interest in how similar the behaviors of two mice, the spiking of two cells, the rates of two stocks, or samples of two signatures are.

The individual-specific spaces will account for the variations due to camera placements, sitting postures, head size/shape, etc. Likewise, the time lag between the two participants may also change depending on the change in the direction of mimicry, complexity of the gesture, etc. In one of our real data illustrations, we have participants switching their roles as leader and follower in the middle of their mimicry session. Thus, analyzing these paired dynamic matrix-variate data requires a strategy that can accommodate two significant challenges: 1) complex multivariate dependence among variables, and 2) dynamic time-varying lags between the two multivariate time series. Here, dynamic time-varying lag refers to the situation when the lag dependency order between the two multivariate time series changes over time. Although our motivating example is from human social interactions, similar challenges are posed by other types of paired multivariate data, such as that collected in animal behavior studies, cellular imaging studies, finance, or speech, gesture, and handwriting recognition.

Joint and Individual Variation Explained (JIVE) (Lock et al., 2013) and Common and Individual Feature Analysis (CIFA) (Zhou et al., 2016) were developed to capture shared and individual-specific features in pairs of multivariate matrices. In the case of JIVE, the data *X*_*i*_’s are decomposed into three parts: a low-rank approximation of joint structure *J*_*i*_, a low-rank approximation of individual variation *S*_*i*_, and an error *E*_*i*_ under the restriction $JS_{i}^{T}=0$ for all *i*. Here *J* is the matrix stacking *J*_*i*_’s on top of each other. The CIFA decomposition defines a matrix factorization problem: ${\text{min}}_{A,A_{i},B_{i},{\tilde{B}}_{i}}{\left\|Y_{i}-{\left(A,A_{i}\right)}^{T}\left(B_{i},{\tilde{B}}_{i}\right)\right\|}_{F}^{2}$ under the restriction that *A*^*T*^ *A*_*i*_ = 0 for all *i*, with ∥·∥_*F*_ denoting the Frobenius norm. Thus, the shared subspace of the data matrix *Y*_*i*_ in the CIFA decomposition is *AB*_*i*_ and the individual specific subspace is characterized by $A_{i}{\tilde{B}}_{i}$. Due to the assumed orthogonality between the columns of *A* and *A*_*i*_, the shared and individual-specific spaces become orthogonal. Extensions of these methods are proposed in Li and Gaynanova (2018) and Feng et al. (2018). Related approaches have been used in behavioral research (Schouteden et al., 2014), genomic clustering (Lock and Dunson, 2013; Ray et al., 2014), railway network analysis (Jere et al., 2014), etc. In most cases, frequentist frameworks are used for inference, the methods are not likelihood-based, and the focus is on static data. De Vito et al. (2021) developed a method for multigroup factor analysis in a Bayesian framework, which has some commonalities with these approaches but does not impose orthogonality.

One way to accommodate time-varying lags is to temporally align the features in a shared space, avoiding the need to develop a complex model of lagged dependence across the series. However, time alignment is a hard problem. Typically, alignment is done in a first stage, and then an inferential model is applied to the aligned data (Vial et al., 2009). However, such two-stage approaches do not provide adequate uncertainty quantification. Trigeorgis et al. (2017) also considered a problem of time aligned image analysis. Their proposed loss function combines costs for non-linear discriminant analysis and dynamic time warping. They further modelled the unknown non-linear functions using deep neural-nets. Unlike our approach, their method does not adjust for individual-specific variations.

Several approaches have been proposed to model warping functions. Tsai et al. (2013) used basis functions similar to B-splines with varying knot positions, using stochastic search variable selection for the knots. This makes the model more flexible, but at the cost of very high computational demand. Kurtek (2017) put a prior on the warping function based on a geometric condition and developed importance sampling methods. Extending their geometric characterization to the multivariate case is not straightforward; hence it is difficult to extend their method to our setting. Lu et al. (2017) use a similar structure in placing a prior on the warping function.

Bharath and Kurtek (2017); Cheng et al. (2016) put a Dirichlet prior on the increments of the warping function over a grid of time points. Thus, the estimated warping function is not smooth. Also, when the warping function is convolved with an unknown function, computation becomes inefficient due to poor mixing. The concept of warplets of Claeskens et al. (2010) is very interesting. Nevertheless, this method also suffers from a similar computational problem.

For multivariate time warping, Listgarten et al. (2005) proposed a method based on a hidden Markov model. Other works propose to use a warping based distance to cluster similar time series (Orsenigo and Vercellis, 2010; Che et al., 2017). Unfortunately, these algorithms require the two time series to be collected at the same time points. In addition, it is difficult to avoid a two-stage procedure, since there is no straightforward way to combine a statistical model with the warping algorithms.

Gervini and Gasser (2004) modeled the warping function as *M*(*t*) = *t* + ∑_*j*_ *s*_*j*_*f*_*j*_(*t*), where *f*_*j*_(*t*)’s are characterized using B-splines with the sum of the *s*_*j*_’s equal to zero. For identifiability, they assumed restrictive conditions on the spline coefficients and did not accommodate multivariate data. Telesca and Inoue (2008) developed a related Bayesian approach, but their structure makes it difficult to apply gradient-based MCMC, and finding a good proposal for efficient sampling is problematic.

We propose to estimate the similarity between two multivariate time series with time-varying lags using a Bayesian dynamic factor model that incorporates time warping and parameter estimation in a single step. Our proposed dynamic factor model is different from traditional state-space models (Aguilar and West, 2000). Instead of assuming any Markovian propagation of the latent factors, we assume the latent factors to vary smoothly over time *t*. We further assume the multivariate time series have both time-aligned shared factors and individual-specific factors. Estimating the shared factors is to assess similarity between the time series, while the main goal of the individual factors is to ensure the inference is robust. The resulting model reduces to a CIFA-style dependence structure, but unlike previous work, we accommodate time dependence and take a Bayesian approach to inference. Key aspects of our Bayesian implementation include likelihood-based estimation of shared and individual-specific subspaces, incorporation of a monotonicity constraint on the warping function for identifiability, and development of an efficient gradient-based Markov chain Monte Carlo (MCMC) algorithm for posterior sampling.

We align the two time series by mapping the features of the shared space using a monotone increasing warping function *M* : [0, 1] → [0, 1]. If we have two univariate time-varying processes *a*(*t*) and *b*(*t*), then the warping function *M* is generally computed as the minimizer of *d*(*a*(*t*), *b*(*M*(*t*))) for some distance metric *d*. To ensure identifiability of *M* in this minimization problem, we need to further assume that *M*(0) = 0, *M*(1) = 1 and *M*(*t*) is monotone increasing. This flexible function *M*(*t*) can accommodate situations where the time lags between the multivariate time series change sign and direction. Our monotone function construction differs from previous Bayesian approaches (Ramsay et al., 1988; He and Shi, 1998; Neelon and Dunson, 2004; Shively et al., 2009; Lin and Dunson, 2014), motivated by tractability in obtaining a nonparametric specification amenable to Hamiltonian Monte Carlo (HMC) sampling.

In general, posterior samples of the loading matrices are not interpretable without identifiability restrictions (Seber, 2009; Lopes and West, 2004; Ročková and George, 2016; Fruehwirth-Schnatter and Lopes, 2018). To avoid arbitrary constraints, which complicate computation, one technique is to post-process an unconstrained MCMC chain. Aßmann et al. (2016) post-process by solving an Orthogonal Procrustes problem to produce a point estimate of the loading matrix, but without uncertainty quantification. We consider to post-process the MCMC chain iteratively so that it becomes possible to draw inference based on the whole chain. Apart from the computational advantages, we also show identifiability of the warping function in our factor modeling setup both in theory and simulations. Moreover, our identifiability result is more general than the result in Gervini and Gasser (2004) as we do not assume any particular form of the warping function other than monotonicity and also it has been derived in a multivariate setting.

In section 2 we discuss our model in detail. Prior specifications are described in Section 3. Our computational scheme is outlined in Section 4. Section 5 discusses theoretical properties such as identifiability of the warping function and posterior concentration. We study the performance of our method in two simulation setups in Section 6. Section 7 considers applications to human social interaction datasets. We end with some concluding remarks in Section 8. Supplementary Materials have all the proofs, additional algorithmic details, and additional results.

## 2. Modeling

We have a pair of *p* dimensional time varying random variables **x**_*t*_ and **y**_*t*_. We propose to model the data as a function of time varying shared latent factors, ***η***(*t*) = {*η*_1_(*t*), …, *η*_*r*_(*t*)}, and individual-specific factors, $\zeta_{1}(t)=\left\{\zeta_{11}(t),\ldots ,\zeta_{1r_{1}}(t)\right\}$ and $\zeta_{2}(t)=\left\{\zeta_{21}(t),\ldots ,\zeta_{2r_{2}}(t)\right\}$. We do time alignment through the shared factors in *η*(*t*) using warping functions *M*_1_(*t*), …, *M*_*r*_(*t*). Here *M*_*i*_ is the warping function for the latent variable *η*_*i*_.

Latent factor modeling is natural in this setting in relating the measured multivariate time series to lower-dimensional characteristics, while reducing the number of parameters needed to represent the covariance. Since we are using the warping function to align the time-varying factors of the shared space, to ensure identifiability, the individual-specific space and the shared space are required to be orthogonal. Thus, the corresponding loading matrices of the two orthogonal subspaces are assumed to have orthogonal column spaces. Let **Λ** be the loading matrix of the shared space. Then the shared space signal belongs to the span of the columns of **Λ** with weights as some multiple of the shared factors ***η***(*t*) = {*η*_1_(*t*), …, *η*_*r*_(*t*)}. An element from the time-varying shared space can be represented as $\sum_{j=1}^{r}a_{j}\Lambda_{.j}\eta_{j}(t)$ for some constant $\left(a_{1},\ldots ,a_{r}\right)\in {\mathbb{R}}^{r}$ where **Λ**_.*j*_ is the *j*-*th* column of **Λ**. Alternatively it can also be written as **ΛΞ**_1_*β*(*t*), where **Ξ**_1_ is a diagonal matrix with entries (*a*_1_, …, *a*_*r*_). The individual-specific space is assumed to be in the orthogonal subspace of the column space of **Λ**. Thus we use the orthogonal projection matrix **Ψ** = **1** − **Λ**(**Λ**^*T*^ **Λ**)^−1^**Λ**^*T*^ to construct the loading matrix of the individual-specific part of each signal. The loading matrix for the individual-specific space **x**_*t*_ is assumed to be **ΨΓ**_1_ for some matrix **Γ**_1_ of dimension *p* × *r*_1_, where *r*_1_ is the rank. The corresponding loading matrix for the individual-specific space of **y**_*t*_ is **ΨΓ**_2_, with **Γ**_2_ being a *p* × *r*_2_ matrix with *r*_2_ the rank. The shared signals of **x**_*t*_ and **y**_*t*_ are **Λ*η***(*t*) and **Λ*η***_1_(*t*). In order to align the two shared spaces, we further assume that the factors in ***η***_1_(*t*) are a warped version of the factors in ***η***(*t*). For simplicity, we assume that there is a single warping function that holds for all the latent factors.

**Figure 1:**
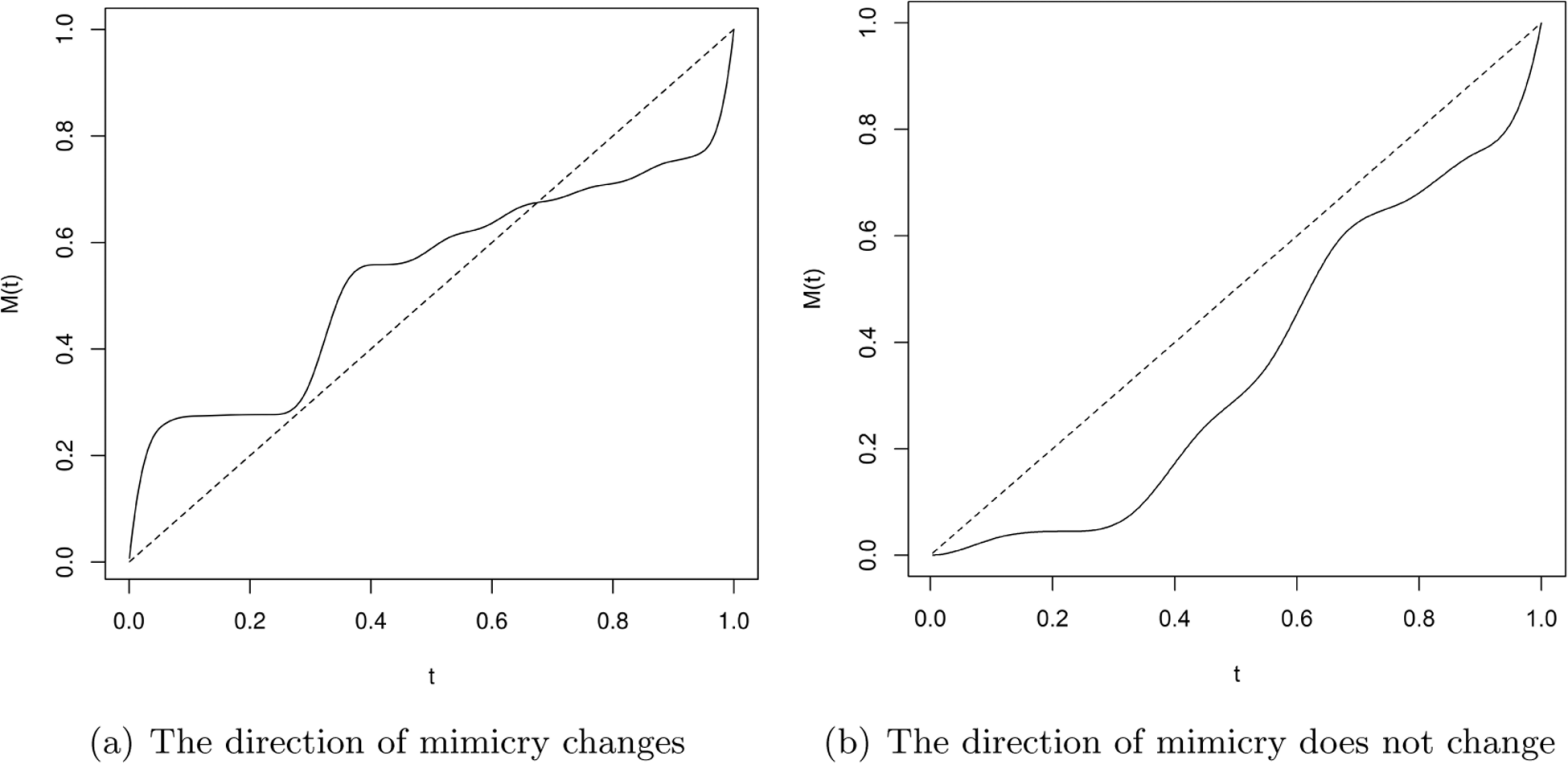
Estimated warping functions for two social mimicry experiments (solid lines). The dashed line is when individual 1 has perfectly aligned behaviors as individual 2.

The warping function *M* : [0, 1] → [0, 1] is assumed to be monotone increasing, which is important for identifiability. As motivation, consider the case of social interactions. People often imitate each other subconsciously. In a normal conversation, people take turns mimicking each other without knowing it. Let us assume that A and B are playing a game where they take turns mimicking each other so that sometimes A mimics B and sometimes B mimics A. This motivates us to model this mimicry to assess how similar A and B’s gestures are. By the definition of a warping function, if person A makes a gesture at time *t*, person B does the same gesture at *M*(*t*). If one person mimics the other almost instantly, we must have *t* = *M*(*t*). Hence, in Figure 1, the dashed line through the origin with slope one corresponds to the case when there is no lag among the participants. However such instantaneous mimicry is often unrealistic. Thus it might be either *t < M*(*t*) or *t* > *M*(*t*) depending on whether individual A or B makes the gesture for the first time. A method that models this mimicry would need to be able to account for the fact that the roles change dynamically over time. In Figure 1, we illustrate behavior of the warping function in two possible experimental situations that we consider in our real data illustration. Hence, panel (a) shows the warping function when one individual is mimicking the other for the first part of the experiment, and then the leader shifts. In the panel (b) experiment, the leader remains the same throughout. Both of these functions are estimated based on real data.

To model a smooth monotone increasing warping function bounded in [0, 1] such that *M*(0) = 0 and *M*(1) = 1 we use a B-spline expansion with *J* many bases as follows,

$$M(t)=\sum_{j=1}^{J}\gamma_{j}B_{j}(t),\gamma_{ij}=\frac{\sum_{\ell =2}^{j}\text{exp}\left(\kappa_{\ell }\right)}{\sum_{k=2}^{J}\text{exp}\left(\kappa_{k}\right)},\;\gamma_{1}=0,$$

where *B*_*j*_(·)’s are B-spline basis functions and *κ*_*k*_ ∈ (−*∞*, *∞*). To restrict *M*(*t*) to be monotone increasing and bounded between [0, 1], it is sufficient to have the B-spline coefficients ${\left\{\gamma_{j}\right\}}_{j=1}^{J}$ be monotone increasing in index *j* and bounded between [0, 1] (De Boor, 1978). This construction restricts *M* to be a smooth monotone increasing function such that *M*(0) = 0 and *M*(1) = 1. These are the desired properties of a warping function. A short review on B-splines is provided in Section 1 of the supplementary materials.

For simplicity, we consider a single warping function for all the shared latent variables. The complete model that we consider is

(1) $$x_{t}=\Psi \Gamma_{1}\zeta_{1}(t)+\Lambda \Xi_{1}\eta (t)+\epsilon_{1t},$$

(2) $$y_{t}=\Psi \Gamma_{2}\zeta_{2}(t)+\Lambda \Xi_{2}\eta (M(t))+\epsilon_{2t},$$

(3) $$\zeta_{ij}(t)=\sum_{j=1}^{K_{i}}\beta_{ilj}B_{j}(t),\;i=1,2;j=1,\ldots r_{i},$$

(4) $$\eta_{i}(t)=\sum_{j=1}^{K}\beta_{ij}B_{j}(t),$$

(5) $$M(t)=\sum_{j=1}^{J}\gamma_{j}B_{j}(t),$$

(6) $$\gamma_{j}=\frac{\sum_{l=2}^{j}\text{exp}\left(\kappa_{l}\right)}{\sum_{k=2}^{J}\text{exp}\left(\kappa_{k}\right)},\;\gamma_{1}=0,$$

(7) $$\epsilon_{it}\sim \text{N}\left(0,\Sigma_{i}\right),\;\Sigma_{i}=\text{diagonal}\left(\sigma_{i1}^{2},\ldots ,\sigma_{ip}^{2}\right),$$

where **Λ**, **Γ**_1_, **Γ**_2_ are static factor loading matrices of dimension *p* × *r*,*p* × *r*_1_ and *p* × *r*_2_, respectively, with **Ψ** = **I**_*p*_ − **Λ**(**Λ**^*T*^ **Λ**)^−1^**Λ**^*T*^; **Ξ**_1_ and **Ξ**_2_ are *r* × *r* diagonal matrices; *r* is the number of shared time varying latent factors and *r*_1_, *r*_2_ are the number of individual-specific latent factors for the 1st and 2nd individual, respectively; the error variances are given by **Σ**_1_ and **Σ**_2_. In (1) and (2), we define ***η***(*t*) = {*η*_*i*_(·) : 1 ≤ *i* ≤ *r*} as the vector of shared time-varying factors. Similarly, we define the individual-specific array of time-varying factors ***ζ***_1_(*t*) = {*ζ*_1*j*_(·) : 1 ≤ *j* ≤ *r*_1_} and ***ζ***_2_(*t*) = {*ζ*_2*j*_(·) : 1 ≤ *j* ≤ *r*_2_}. In (3), we denote the number of B-spline bases to model individual-specific factors of the *i*-*th* individual by *K*_*i*_. To model the shared time-varying latent factors, *η*_*i*_(·)’s, we use *K* B-spline bases in (4). The number of bases to model the warping function in (5) is *J*. The constraint *γ*_1_ = 0 ensures *M*(0) = 0 and the softmax type reparametrization ensures monotonicity. Under the above characterization, we have $\eta_{i}(M(t))=\sum_{j=1}^{K}\beta_{ij}B_{j}\left\{\sum_{\ell =1}^{J}\gamma_{\ell }B_{\ell }(t)\right\}$.

**Figure 2:**
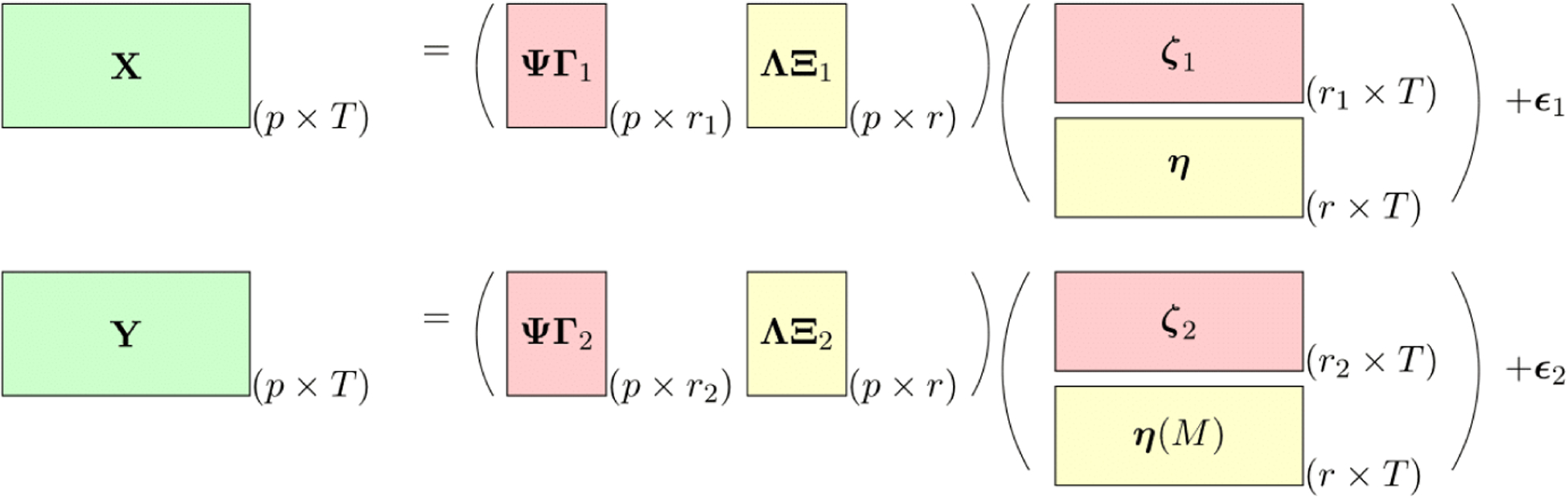
A schematic representation of our proposed model where the dimensions of the matrices are illustrated at the bottom right corner, and ***η***(*M*) stands for time aligned factors from ***η*** using the warping function *M*(*t*) and **Ψ** = **I**_*p*_ − **Λ**(**Λ**^*T*^ **Λ**)^−1^**Λ**^*T*^. The dimensions of the individual matrices, **Λ**, **Γ**_1_, **Γ**_2_ are *p* × *r*,*p* × *r*_1_ and *p* × *r*_2_ respectively. The other two matrices **Ξ**_1_ and **Ξ**_2_ are *r* × *r* diagonal matrices. Additionally, in the Figure, **X** = [**x**_1_; ⋯ ; **x**_*T*_],**Y** = [**y**_1_; ⋯ ; **y**_*T*_], ***ζ***_1_ = [***ζ***_1_(1); ⋯ ; ***ζ***_1_(*T*)],***ζ***_2_ = [***ζ***_2_(1); ⋯ ; ***ζ***_2_(*T*)] and ***η*** = [***η***(1); ⋯ ; ***η***(*T*)]

A schematic representation of our proposed model is shown in Figure 2. We project the individual-specific loading matrices on the orthogonal space of the shared space spanned by columns of **Λ** using **Ψ**. The data are collected over *T* time points longitudinally for individual 1 and 2 respectively, and *X* and *Y* are *p* × *T* and *p* × *T* dimensional data matrices. Correspondingly, **ΨΓ**_1_***ζ*** and **ΛΞ**_1_*β* are the individual-specific mean and shared space mean of *X*, respectively. The columns of these two matrices are orthogonal due to the orthogonality of **Ψ** and **Λ**. Since ***ζ***_1_(*t*) and ***η***(*t*) are modeled independently, the rows of the two means are also independent in probability. A similar result holds for *Y*. Thus, this model conveniently explains both joint and individual variations.

The loading matrix **Λ** identifies the shared space of the two signals. We assume a single shared set of latent factors ***η***(*t*) for both *X*_*t*_ and *Y*_*t*_. The warping function *M*(*t*) aligns those for the *Y*_*t*_ series relative to the **x**_*t*_ series. Then we have individual-specific factors ***ζ***_1_(*t*), ***ζ***_2_(*t*) and factor loading matrices **ΨΓ**_1_, **ΨΓ**_2_ that can accommodate within series covariances in **x**(*t*) and **y**(*t*). We call our proposed method Time Aligned Common and Individual Factor Analysis (TACIFA).

## 3. Prior specification

We use priors similar to those in Bhattacharya and Dunson (2011) for **Λ**, **Γ**_1_ and **Γ**_2_ to allow for automatic selection of rank. We try to maintain conjugacy as much as possible for easier posterior sampling. For clarity, we define ***κ*** = {*κ*_*j*_ : 2 ≤ *j* ≤ *J* } and ***β*** = {*β*_*ij*_ : 1 ≤ *j* ≤ *r*_*i*_, 1 ≤ *i* ≤ 2, } The detailed prior description for ***κ***, ***β***, **Λ**, **Ξ**_1_, **Ξ**_2_, **Γ**_1_, **Γ**_2_, ***σ***_1_ and ***σ***_2_ is described below,

(8) $$\Lambda_{lk}|\phi_{1,lk},\tau_{1k}\sim \text{N}\left(0,\phi_{1,lk}^{-1}\tau_{1k}^{-1}\right),\;1\leq l\leq p,1\leq k\leq r,$$

(9) $$\phi_{1,lk}\sim \text{Gamma}\left(\nu_{1},\nu_{1}\right),\;\tau_{1k}=\prod_{i=1}^{k}\delta_{mi},\;1\leq l\leq p,1\leq k\leq r,$$

(10) $$\delta_{1,1}\sim \text{Gamma}\left(\alpha_{1},1\right),\;\delta_{1,i}\sim \text{Gamma}\left(\alpha_{2},1\right),1\leq i\leq r,$$

(11) $$\Gamma_{1,lk}|\phi_{11,lk},\tau_{11k}\sim \text{N}\left(0,\phi_{11,lk}^{-1}\tau_{11k}^{-1}\right),\;1\leq l\leq p,1\leq k\leq r_{1},$$

(12) $$\phi_{11,lk}\sim \text{Gamma}\left(\nu_{1},\nu_{1}\right),\;\tau_{11k}=\prod_{i=1}^{k}\delta_{mi}$$

(13) $$\delta_{11,1}\sim \text{Gamma}\left(\alpha_{111},1\right),\;\delta_{11,i}\sim \text{Gamma}\left(\alpha_{112},1\right),$$

(14) $$\Gamma_{2,lk}|\phi_{12,lk},\tau_{12k}\sim \text{N}\left(0,\phi_{12,lk}^{-1}\tau_{12k}^{-1}\right),\;1\leq l\leq p,1\leq k\leq r_{2},$$

(15) $$\phi_{12,lk}\sim \text{Gamma}\left(\nu_{1},\nu_{1}\right),\;\tau_{12k}=\prod_{i=1}^{k}\delta_{mi},$$

(16) $$\delta_{12,1}\sim \text{Gamma}\left(\alpha_{121},1\right),\;\delta_{12,i}\sim \text{Gamma}\left(\alpha_{122},1\right),$$

(17) $$\sigma_{1l}^{-2}\sim \text{Gamma}\left(\alpha_{1},\alpha_{1}\right),\;\sigma_{2l}^{-2}\sim \text{Gamma}\left(\alpha_{2},\alpha_{2}\right),\;1\leq l\leq p$$

(18) $$\Xi_{1,ll},\Xi_{2,ll},\kappa_{j},\beta_{qk}\beta_{siK_{s}}\sim N(0,\omega ),$$

for 1 ≤ *k* ≤ *K*, *q* = 1, …, *r* 1 ≤ *j* ≤ *J*, *i* = 1, …, *r*_*s*_, *s* = 1, 2 and *l* = 1, … , *r*. Higher values of *α*_*m*2_ ensure increasing shrinkage as we increase rank.

We initially set the number of factors to a conservative upper bound. Then the multiplicative gamma prior will tend to induce posteriors for $\tau_{k}^{-1}$ in the later columns that are concentrated near zero. Those columns in **Λ** will tend to zero. Thus, the corresponding factors are then effectively deleted. The extra factors in the model may either be left, as they will have essentially no impact, or may be removed via a factor selection procedure which will remove the columns having entries within ±*ζ* of zero. We follow the second strategy, motivated by our goal of obtaining a few interpretable factors. In particular, we apply the adaptive MCMC procedure of Bhattacharya and Dunson (2011) with *ζ* = 1 × 10^−3^.

## 4. Computation

We use Gibbs updates for all the parameters except for **Λ** and ***κ***; details are provided in Section 2 of Supplementary Materials. For **Λ** and ***κ***, we propose an efficient gradient-based MCMC algorithm. For our proposed model, we can easily calculate the derivative of the log-likelihood with respect to ***κ*** using derivatives of B-splines (De Boor, 1978). This parameter ***κ*** is only involved in the model of **y**_*t*_. The negative of that log-likelihood function including the prior on ***κ*** is

$$L(\kappa )=\sum_{t=1}^{T}\sum_{i=1}^{p}\frac{1}{\sigma_{2i}^{2}}{\left[Y_{it}-\Psi_{2i}\zeta_{2}(t)-\Lambda_{2i}\eta \left\{\sum_{j=1}^{J}\frac{\sum_{l=2}^{j}\text{exp}\left(\kappa_{l}\right)}{\sum_{k=2}^{J}\text{exp}\left(\kappa_{k}\right)}B_{j}(t)\right\}\right]}^{2}+\frac{\sum_{j=2}^{J}\kappa_{j}^{2}}{2\omega^{2}}.$$

For simplicity in expression of the derivative, let us denote $A_{it}=\Lambda_{2i}\eta \left(\sum_{J=1}^{J}\frac{\sum_{l=2}^{j}\text{exp}\left(\kappa_{l}\right)}{\sum_{k=2}^{J}\text{exp}\left(\kappa_{k}\right)}B_{j}(t)\right)$ and $M(t)=\sum_{J=1}^{J}\frac{\sum_{l=2}^{j}\text{exp}\left(\kappa_{l}\right)}{\sum_{k=2}^{J}\text{exp}\left(\kappa_{k}\right)}B_{j}(t)$, as defined earlier. Then the derivative is given by

$$L^{\prime }\left(\kappa_{j}\right)=-\sum_{t=1}^{T}\sum_{i=1}^{p}\frac{1}{\sigma_{2i}^{2}}\left(Y_{it}-\Psi_{2i}\zeta_{2}(t)-A_{it}\right)A_{it}\left[\sum_{l=j}^{J}B_{l}(t)-M(t)\right]\text{exp}\left(\kappa_{j}\right)/\sum_{k=2}^{J}\text{exp}\left(\kappa_{k}\right)+\kappa_{j}/\omega^{2}.$$

Let us denote *L*′(***κ***) = (*L*′(*κ*_2_), …, *L*′(*κ*_*J*_))′.

Now, we discuss the sampling for Λ. To update the *j*-*th* column of Λ, we first rewrite the orthogonal projection matrix using the matrix inverse result of block matrices as

$$\Psi =\left(1-P_{1}\right)\left(1-P_{2}\right)\left(1-P_{1}\right)$$

where $P_{1}=\Lambda_{.-j}{\left(\Lambda_{-j}^{T}\Lambda_{-j}\right)}^{-1}\Lambda_{.-j}^{T}$ and $P_{2}=\Lambda_{.j}{\left(\Lambda_{j}^{T}\left(1-P_{1}\right)\Lambda_{j}\right)}^{-1}\Lambda_{.j}^{T}$. Here **Λ**_.−*j*_ is the reduced matrix after removing the *j*-*th* column of **Λ** and **Λ**_.*j*_ is the *j*-*th* column. The negative log-likelihood with respect to **Λ**_.*j*_ is

$$L_{1}\left(\Lambda_{.j}\right)=\sum_{t}\sum_{i=1}^{p}{\left(X-\Psi \Gamma_{1}\zeta_{1}(t)-\Lambda \Xi_{1}\eta (t)\right)}^{2}/\left(2\sigma_{1}^{2}\right)\;+\sum_{t}\sum_{i=1}^{p}{\left(Y-\Psi \Gamma_{2}\zeta_{1}(t)-\lambda \Xi_{2}\eta (t)\right)}^{2}/\left(2\sigma_{2}^{2}\right)+\sum_{k}\Lambda_{kj}^{2}/\left(2\phi_{1,kj}\tau_{j}\right),$$

and the derivative is

$$L_{1}^{\prime }\left(\Lambda_{kj}\right)=\sum_{t}\sum_{i=1}^{p}\left(X_{ti}-{\Psi \Gamma }_{1}\zeta_{1}(t)-\Lambda \Xi_{1}\eta (t)\right)\left(B_{ti}-\eta (t)\right)/\left(\sigma_{1}^{2}\right)+\sum_{t}\sum_{i=1}^{p}(Y_{ti}-\Psi \Gamma_{2}\zeta_{2}(t)-\Lambda \Xi_{2}\eta (M(t)))\left(B_{ti}-\eta (t)\right)/\left(\sigma_{2}^{2}\right)+\Lambda_{kj}/\left(\phi_{1,kj}\tau_{j}\right),$$

where

$$B=-\left(1-P_{1}\right)Q\left(1-P_{1}\right)$$

$$Q=\{(\left(\Lambda_{.j}e_{k}^{T}+e_{k}\Lambda_{.j}^{T}\right)\Lambda_{.j}^{T}\left(1-P_{1}\right)\Lambda_{.j}\;-2e_{k}\left(1-P_{1}\right)\Lambda_{.j}^{T}\Lambda_{.j}\left(1-P_{1}\right)\Lambda_{.j}^{T})/{\left(\Lambda_{.j}^{T}\left(1-P_{1}\right)\Lambda_{.j}\right)}^{2}\},$$

with **e**_*k*_ a vector of length *p* having 1 at the *k*-*th* position and zero elsewhere.

Relying on the above gradient calculations we use HMC (Duane et al., 1987; Neal et al., 2011). We keep the leapfrog step fixed at 30. We tune the step size parameter to maintain an acceptance rate within the range of 0.6 to 0.8. If the acceptance rate is less than 0.6, we reduce the step length and increase it if the acceptance rate is more than 0.8. We do this adjustment after every 100 iterations. We also incorporate removal of columns of **Λ**, **Γ**_1_ and **Γ**_2_ if the contributions are below a certain threshold as described in Section 3.2 of Bhattacharya and Dunson (2011).

### 4.1 Post-MCMC inference

Here we discuss the strategy to infer the loading matrix **Λ**_1_ = **ΛΞ**_1_. The loading matrices are identifiable up to an orthogonal right rotation. This implies that (**Λ**_1_, ***η***(*t*)) and (**Λ**_1_**R**, **R**^*T*^ ***η***(*t*)) for some orthonormal matrix *R* have equivalent likelihood. In our modeling framework, we may write ***η***(*t*) = ***β*B**_*t*_, where ***β*** = ((*β*_*ij*_))_1≤*i*≤*r*,1≤*j*≤*K*_ is the coefficient matrix and **B**_*t*_ = (*B*_1_(*t*), …, *B*_*K*_(*t*)) is the array of *K* B-spline bases evaluated at *t*. Thus, **R**^*T*^ ***η*** gives us a new array of latent factors with coefficient matrix **R**^*T*^ ***β***. However, the same likelihood is obtained for values of (**Λ**_1_, ***η***(*t*)) or (**Λ**_1_**R**, **R**^*T*^ ***η***(*t*)), implying non-identifiability.

Let $\Lambda_{1}^{\left(1\right)},\ldots ,\Lambda_{1}^{\left(m\right)}$ be *m* post burn-in samples of Λ_1_. To address the non-identifiability problem, we post-process the chain successively moving from the first sample to the last. First $\Lambda_{1}^{\left(2\right)}$ is rotated with respect to $\Lambda_{1}^{\left(1\right)}$ using some orthonormal matrix **R**_1_ such that ${\left\|\Lambda_{1}^{\left(1\right)}-\Lambda_{1}^{\left(2\right)}R_{1}\right\|}_{F}^{2}$ is minimized, where ${\left\|\right\|}_{F}^{2}$ denotes the Frobenius norm. This minimization criterion rotates $\Lambda_{1}^{\left(2\right)}$ to make it as close as possible to $\Lambda_{1}^{\left(1\right)}$. The solution of **R**_1_ is obtained in Theorem 1. Then we post-process $\Lambda_{1}^{\left(3\right)}$ with respect to $\Lambda_{1}^{\left(2\right)}R_{1}$ and so on.

**Theorem 1** *The minimizer* **R**_1_ *of the objective function ${\left\|\Lambda_{1}^{\left(1\right)}-\Lambda_{1}^{\left(2\right)}R_{1}\right\|}_{F}^{2}$ is given by $R_{1}=Q_{2}Q_{1}^{T}$, where $Q_{1}DQ_{2}^{T}$ is the singular value decomposition (SVD) of* ${\left(\Lambda_{1}^{\left(1\right)}\right)}^{T}\Lambda_{1}^{\left(2\right)}$.

The proof of the theorem is in the Section 1.1 of Supplementary Materials. Intuitively, the columns of **Q**_1_ and **Q**_2_ are the canonical correlation components of $\Lambda_{1}^{\left(1\right)}$ and $\Lambda_{1}^{\left(2\right)}$, respectively. Thus the rotation matrix **R**_1_ rotates $\Lambda_{1}^{\left(2\right)}$ towards the least principal angle between $\Lambda_{1}^{\left(2\right)}$ and $\Lambda_{1}^{\left(1\right)}$. For instance, $\Lambda_{1}^{\left(2\right)}$ could be an exact right rotation of $\Lambda_{1}^{\left(1\right)}$. Thus before starting to post-process the MCMC chain, we transform $\Lambda_{1}^{\left(1\right)}$ as $\Lambda_{1}^{\left(1\right)}U_{2}$ such that $U_{1}EU_{2}^{T}$ is the SVD of the residual ${\left(x_{t}-\Psi^{\left(1\right)}\Gamma_{1}^{\left(1\right)}\zeta {\left(t\right)}^{\left(1\right)}\right)}^{T}\Lambda_{1}^{\left(1\right)}$ in the same way and here *E* is the diagonal matrix with elements in decreasing order. This initial transformation ensures that the higher order columns of the loading matrix are lower in significance in explaining the data. Then following the above result, we post-process the rest of the MCMC chain of the loading matrix on the post burn-in samples successively. In general, SVD computation is expensive. However, in most applications, the estimated rank is very small. Thus the computation becomes manageable. After the post-processing, we can construct credible bands for the parameters. We apply this post-processing step for all the loading matrices.

### 4.2 Measure of similarity

It is of interest to quantify similarity between paired time series. We propose the following measure of similarity,

$$\text{Syn}(X,Y)=1-\frac{1}{pT}\sum_{l}\left|\sum_{t}\left[\frac{{\left(\Lambda_{l}\Xi_{1}\eta (t)\right)}^{2}}{{\left(\Psi_{l}\Gamma_{1}\zeta_{1}(t)\right)}^{2}+{\left(\Lambda_{l}\Xi_{1}\eta (t)\right)}^{2}+\sigma_{1l}^{2}}-\frac{{\left(\Lambda_{l}\Xi_{2}\eta (M(t))\right)}^{2}}{{\left(\Psi_{l}\Gamma_{2}\zeta_{2}(t)\right)}^{2}+{\left(\Lambda_{l}\Xi_{2}\eta (M(t))\right)}^{2}+\sigma_{2l}^{2}}\right]\right|,$$

where **Λ**_*l*_, **Ψ**_*l*_ denote the *l*^*th*^ row of the corresponding matrices and *p*,*T* denote number of features and time points respectively. The measure ‘Syn’ is bounded between [0, 1]. Here, the difference in relative contribution of each feature on the two shared spaces is considered as a measure of dissimilarity. Then as a measure of similarity, we consider the difference of the average dissimilarity from one. Smaller Syn-value would suggest that the warping function is not able to align the shared space perfectly.

## 5. Theoretical support

In this section, we provide some theoretical justification for our model. Identifiability of the warping function is a desirable property as well as posterior consistency.

### 5.1 Identifiability of the warping function

The following result shows that the warping function *M*(*t*) is identifiable for model (2).

**Theorem 2** *The warping function M*(*t*) *is identifiable if η*(*t*) *is continuous and not constant at any interval of time*.

The proof is by contradiction. Details of the proof are in Section 1.2 of Supplementary Materials. The assumptions on *η*(*t*) are very similar to those assumed for the ‘structural mean’ in Gervini and Gasser (2004). The continuity assumption of *η*(*t*) can be replaced with a ‘piecewise monotone without flat parts’ assumption (Gervini and Gasser, 2004). The proof is still valid with minor modifications for this alternative assumption. In our model *η*(*t*) is varying with time smoothly. Thus *M*(*t*) is identifiable.

### 5.2 Asymptotic result

We study the posterior consistency of our proposed model. Our original model is

(19) $$x_{t}=\Psi \Gamma_{1}\zeta_{1}(t)+\Lambda \Xi_{1}\eta (t)+\epsilon_{1t},\;\epsilon_{1t}\sim \text{N}\left(0,\sigma_{1}^{2}\right),\;y_{t}=\Psi \Gamma_{2}\zeta_{2}(t)+\Lambda \Xi_{2}\eta (M(t))+\epsilon_{2t},\;\epsilon_{2t}\sim \text{N}\left(0,\sigma_{2}^{2}\right).$$

We first show posterior concentration of a simplified model that drops **Ξ**_1_ and **Ξ**_2_. Then using that result we show posterior concentration of model (19) in Corollary 4. We rewrite ***ζ***_1_(*t*) = **ΨΓ**_1_***ζ***_1_(*t*), ***ζ***_2_(*t*) = **ΨΓ**_2_***ζ***_2_(*t*) and ***η***(*t*) = **Λ*η***(*t*). Based on the constructions, ***ζ***_*i*_(*t*) and ***η***(*t*) are orthogonal for *i* = 1, 2. We consider the following simplified model,

$$x_{t_{i}}=\zeta_{1}\left(t_{i}\right)+\eta \left(t_{i}\right)+\epsilon_{1t_{i}},\;\epsilon_{1t}\sim \text{N}\left(0,\sigma_{1t_{i}}^{2}\right),$$

$$y_{t_{i}}=\zeta_{2}\left(t_{i}\right)+\eta \left(M\left(t_{i}\right)\right)+\epsilon_{2},\;\epsilon_{2t}\sim \text{N}\left(0,\sigma_{2}^{2}\right),$$

for 0 ≤ *t*_*i*_ ≤ 1 and *i* = 1, …, *n*. We study asymptotic properties in the increasing *n* and fixed *p* regime. We need to truncate the B-spline series after a certain level or place a shrinkage prior on the number of B-splines as $\Pi [K=k]=b_{1}^{\prime }\text{exp}\left[-b_{2}^{\prime }\;k{\left(\text{log}\;k\right)}^{b_{3}^{\prime }}\right]$, $\Pi [J=j]=b_{1}\text{exp}\left[-b_{2}j{\left(\text{log}\;j\right)}^{b_{3}}\right]$, $\Pi \left[K_{i}=j\right]=b_{i1}\text{exp}\left[-b_{i2}j{\left(\text{log}\;j\right)}^{b_{i3}}\right]$ for *i* = 1, 2, with *b*_1_, *b*_2_, *b*_12_, $b_{22}b_{1}^{\prime }$, $b_{2}^{\prime }$, *b*_11_, *b*_21_ > 0 and 0 ≤ *b*_3_, $b_{3}^{\prime }$, *b*_13_, *b*_23_ ≤ 1. For *b*_3_ = 0 we obtain a geometric distribution and for *b*_3_ = 1, a Poisson distribution.

To study posterior contraction rates, we consider the empirical *ℓ*_2_-distance on the regression functions. The empirical *ℓ*_2_-distance for the two sets of parameters (***ζ***_11_, ***ζ***_21_, ***η***_1_, *M*_1_) and (***ζ***_12_, ***ζ***_22_, ***η***_2_, *M*_2_) is given by

$$d^{2}\left(\left(\zeta_{11},\zeta_{21},\eta_{1},M_{1}\right),\left(\zeta_{12},\zeta_{22},\eta_{2},M_{2}\right)\right)\;=\frac{1}{n}\sum_{i=1}^{n}[\|\zeta_{11}\left(t_{i}\right)-\zeta_{12}{\left(t_{i}\right)\|}_{2}^{2}+\|\zeta_{21}\left(t_{i}\right)-\zeta_{22}{\left(t_{i}\right)\|}_{2}^{2}+\|\eta_{1}\left(t_{i}\right)-\eta_{2}{\left(t_{i}\right)\|}_{2}^{2}\;+\|\eta_{1}\left(M_{1}\left(t_{i}\right)\right)-\eta_{2}{\left(M_{2}\left(t_{i}\right)\right)\|}_{2}^{2}].$$

The smoothness of the underlying true functions ***ζ***_10_, ***ζ***_20_, *β*_0_ and *M*_0_ plays the most significant role in determining the contraction rate. The fixed dimensional parameters ***σ***_1_ and ***σ***_2_ do not have much impact on the rate. The constants *b*_13_, *b*_23_, *b*_3_ and $b_{3}^{\prime }$ appearing in the prior for the number of B-spline coefficients *K*_1_, *K*_2_, *K*, *J* have a mild effect.

**Theorem 3** *Assume that the true functions* ***ζ***_10_, ***ζ***_20_, ***η***_0_ *and M*_0_ *belong to Hölder classes of smooth functions and are of regularity levels ι*_1_, *ι*_2_, *ι and ι′ on* [0, 1]. *Then the posterior contraction rate is given by*

$$n^{-\bar{\iota }/(2\bar{\iota }+1)}{\left(\text{log}\;n\right)}^{\bar{\iota }/(2\bar{\iota }+1)+\left(1-\bar{b_{3}}\right)/2},$$

*where $\bar{\iota }=\text{min}\left\{\iota ,\iota_{1},\iota_{2},\iota^{\prime }\right\}$ and ${\bar{b}}_{3}=\text{min}\left\{b_{3},b_{3}^{\prime },b_{13},b_{23}\right\}$*.

The proof is based on the general theory of posterior contraction as in Ghosal and Van der Vaart (2017) for non-identically distributed independent observations and results for finite random series priors (Shen and Ghosal, 2015). Details of the proof are in Section 1.3 of Supplementary Materials.

Let the parameter space for dynamic latent factors ***ζ***_1_, ***ζ***_2_, ***η*** be F, which is the class of real-valued smooth continuous functions on [0,1], and for the warping function M be the class of [0, 1] bounded smooth monotone continuous functions on [0,1]. Let $\tilde{X}$, $\tilde{X}$, $\tilde{L}$, ${\tilde{G}}_{1}$, ${\tilde{G}}_{2}$ be the priors for the matrices **Ξ**_1_, **Ξ**_2_, **Λ**, **Γ**_1_, **Γ**_2_, respectively, and X, L, $G_{1}$, $G_{2}$ are the parameter spaces of $\tilde{X}$, $\tilde{L}$, ${\tilde{G}}_{1}$, ${\tilde{G}}_{2}$, respectively.

*Assumption 1*: For the true loading matrices and functions, we have $\left\{\Xi_{10},\Xi_{20},\Lambda_{0},\Gamma_{10},\Gamma_{20},\zeta_{10},\zeta_{20},\beta_{0},M_{0}\right\}\in X^{2}\times L\times G_{1}\times G_{2}\times F^{3}\times M$.

Similarly we can define empirical *ℓ*_2_-distance $d_{1}^{2}(\left(\Psi_{1},\Lambda_{1},\Gamma_{11},\Gamma_{12},\Xi_{11},\Xi_{12},\zeta_{11},\zeta_{21},\eta_{1},M_{1}\right),\;\left(\Psi_{2},\Lambda_{2},\Gamma_{21},\Gamma_{22},\Xi_{21},\Xi_{22},\zeta_{12},\zeta_{22},\eta_{2},M_{2}\right))$ as *d*^2^ for the full model and we have following consistency result.

**Corollary 4** *Under the above assumption*, *the posterior for parameters in the model* (19) *is consistent with respect to the distance d*_1_.

For the full model in (19), the test constructions will remain the same as in the proof of Theorem 3. We only need to verify the Kullback-Leibler prior positivity condition. Within our modeling framework, Assumption 1 trivially holds. Details of the proof are in Section 1.4 of Supplementary Materials. The posterior contraction rate of this full model will be the same as the given rate of Theorem 3 as the loading matrices can at most be *p*×*p*-dimensional and we assume *p* is fixed.

## 6. Simulation Study

We run two simulations to evaluate the performance of TACIFA on pairs of multivariate time series. We evaluate TACIFA by: (1) ability to retrieve the appropriate number of shared and individual factors, (2) accuracy of the estimated warping functions and accompanying uncertainty quantification, (3) out of sample prediction errors, and (4) performance relative to two-stage approaches for estimating shared and individual-specific dynamic factors. In the first simulation, we generate data from the proposed model. In the second simulation, we analyze two shapes changing over time, data that does not have any inherent connection to our proposed model. We add two more simulations in Section 4 of Supplementary Materials. One of these two simulations focus on the case where direction of mimicry is changed. The other one corresponds to the case where there is no mimicry.

To assess out of sample prediction error, we randomly assign 90% of the time-points to the training set and the remaining 10% to the test set. Thus, the training set contains a randomly selected 90% of the columns of the data and the remaining 10% columns will be in the test set. The two-stage approaches we compare our method to apply JIVE on the training set in the first stage to estimate the shared space and warp the shared matrices, and then apply multivariate imputation algorithms (missForest, MICE, mtsdi) in the second stage to make predictions on the testing data set. We evaluate the performance of naive dynamic time warping (based solely on minimization of Euclidean distance), derivative dynamic time warping (based on local derivatives of the time data to avoid singularity points), and sliding window based dynamic time warping. Since our model is the only approach with a mechanism for uncertainty quantification, we can compare the prediction performance of TACIFA to two-stage approaches, but we cannot compare uncertainty estimation.

The individual-specific loading matrices are **ΨΓ**_1_ and **ΨΓ**_2_. The shared space loading matrices are **ΛΞ**_1_ and **ΛΞ**_2_. For the (*i*, *j*)-*th* coordinate of a loading matrix *A*, we define a summary measure *SP*_*i*,*j*_(*A*) = (|0.5 − *P*(*A*[*i*, *j*] > 0)|)/0.5 quantifying the “importance” of the element. Here *P*(*A*[*i*, *j*] > 0) is the posterior probability estimated from the MCMC samples of *A* after performing the post-processing steps defined in Section 4.1. These scores help to quantify the importance of the factors and to estimate the number of important factors retrieved by the model.

### 6.1 Simulation case 1

We generate data from a factor model with the following specifications: *ζ*_1*k*_(*t*) = sin(*kt*), *ζ*_2*k*_(*t*) = cos(*kt*) and *M*_0_(*t*) = *t*^0.5^, with *k* varying from 1 to 10. The shared latent factors *η*_*k*_(*t*)’s are set to *k*-*th* degree orthogonal polynomials using the R function poly. The factor loading matrices are of dimension 15 × 3, with the elements of Γ_1_,Γ_2_ generated independently from N(0, 0.1^2^). The entries in the true Λ are structured as a block diagonal matrix as shown in the first image of Figure 4, where the non-zero entries are generated from N(15, 0.1^2^). We vary *t* from 1/500 to 1 with an increment of 1/500. The data *X*_*t*_ and *Y*_*t*_ are generated from N(**Ψ*ζ***_1_ +**Λ*η***(*t*), 1) and N(**Ψ*ζ***_2_ +**Λ*η***(*M*(*t*)), 1), respectively, where *β*(*t*) = (*η*_1_(*t*), *η*_2_(*t*), *η*_3_(*t*)) and **Ψ** = **1** − **Λ**(**Λ**^*T*^ **Λ**)^−1^**Λ**^*T*^.

The choices of hyper parameters are *ω* = 100, *α*_*i*1_ = *α*_*i*2_ = 5 for *i* = 1, 2. We set *K*_1_ = *K*_2_ = *J* = *K* and fit the model for 4 different choices of *K* = 6, 8, 10, 12. The choice *K* = 10 yields the best results among all the candidates. The hyperparameters of the inverse gamma priors for the variance components are all 0.1 which is weakly informative. We collect 6000 MCMC samples and consider the last 3000 as post burn-in samples for inferences. We start the MCMC chain setting the number of shared latent factors *r* = *p* as a very conservative upper bound.

**Figure 3:**
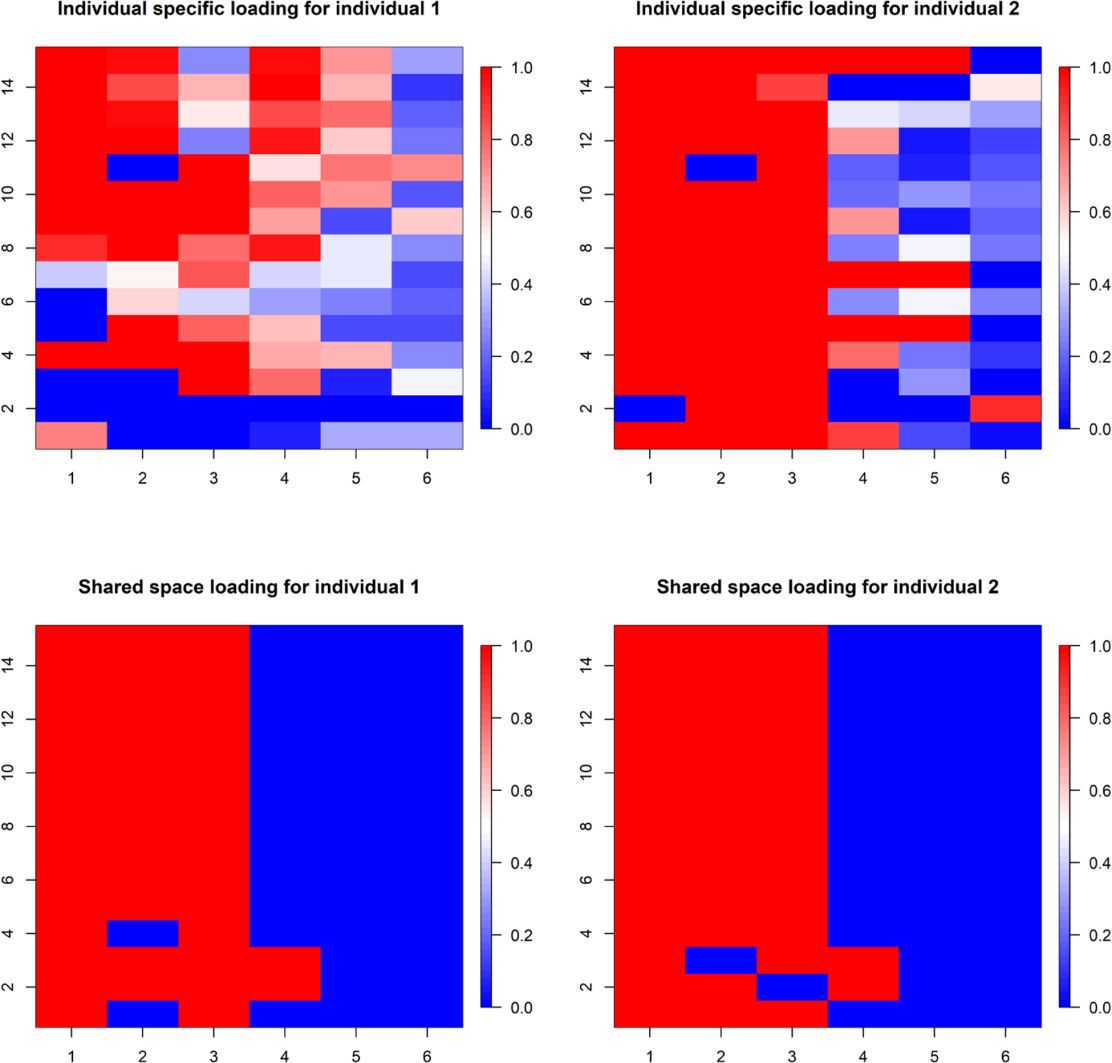
Estimated importance measures *SP* for loading matrices of shared and individual spaces of Series 1 and 2 in Simulation Case 1. Each column represents each factor. The columns with higher proportion of red correspond to the factors with higher importance.

First, we evaluate whether our model retrieves the appropriate number of factors. The true dimension of Λ is 15 × 3. Figure 3 suggests that TACIFA retrieves 3 important shared space factors, as expected. The individual-specific loading matrices in Figure 3 also suggest approximately three important factors.

**Figure 4:**
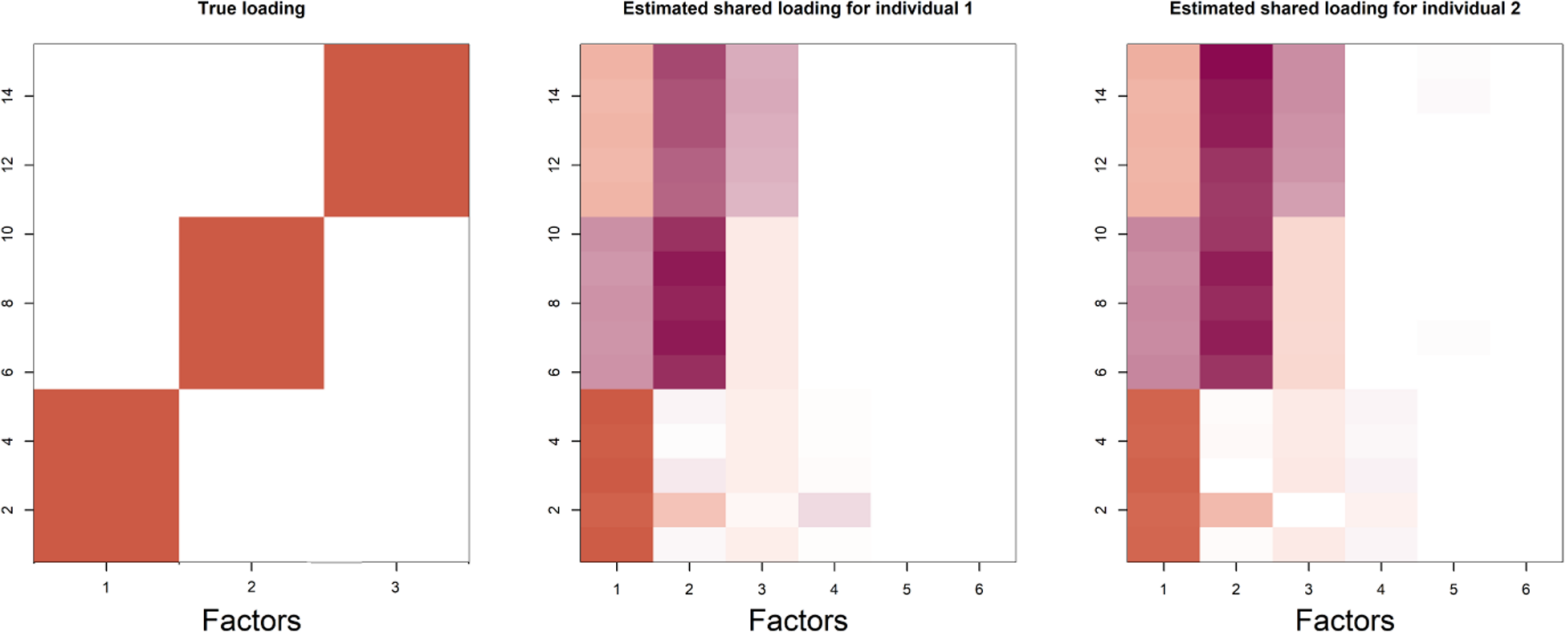
Estimated shared loading matrices along with the true loading structure in Simulation Case 1.

**Figure 5:**
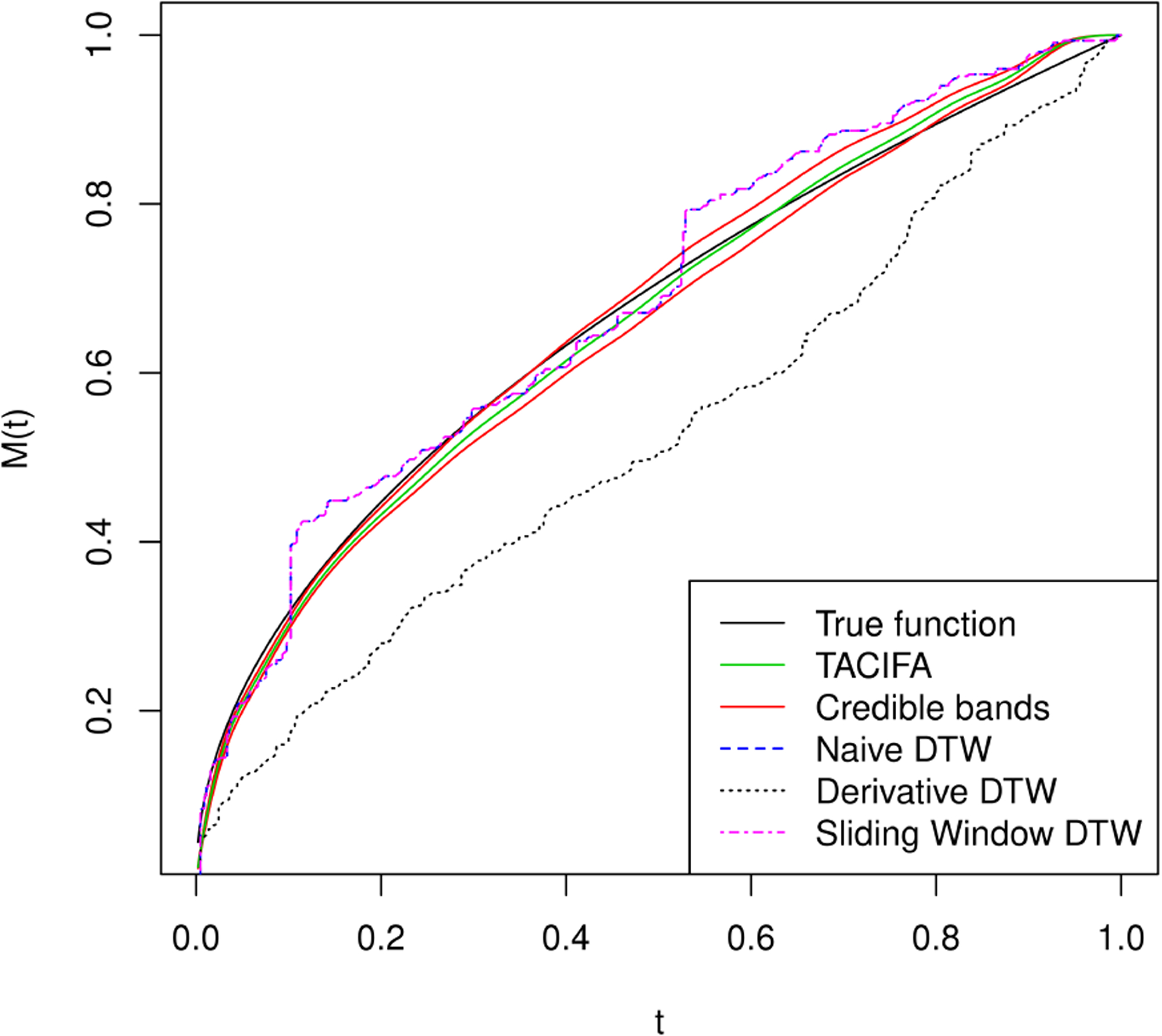
Estimated warping function for simulated data in Simulation Case 1. The black curve is the true warping function *M*_0_(*t*) = *t*^0.5^, the green curve is the estimated function, 95% credible bands are shown in red. Naive DTW and Sliding window DTW curves are indistinguishable. Of all the methods tested, the TACIFA estimated warping function is closest to the true warping function.

**Figure 6:**
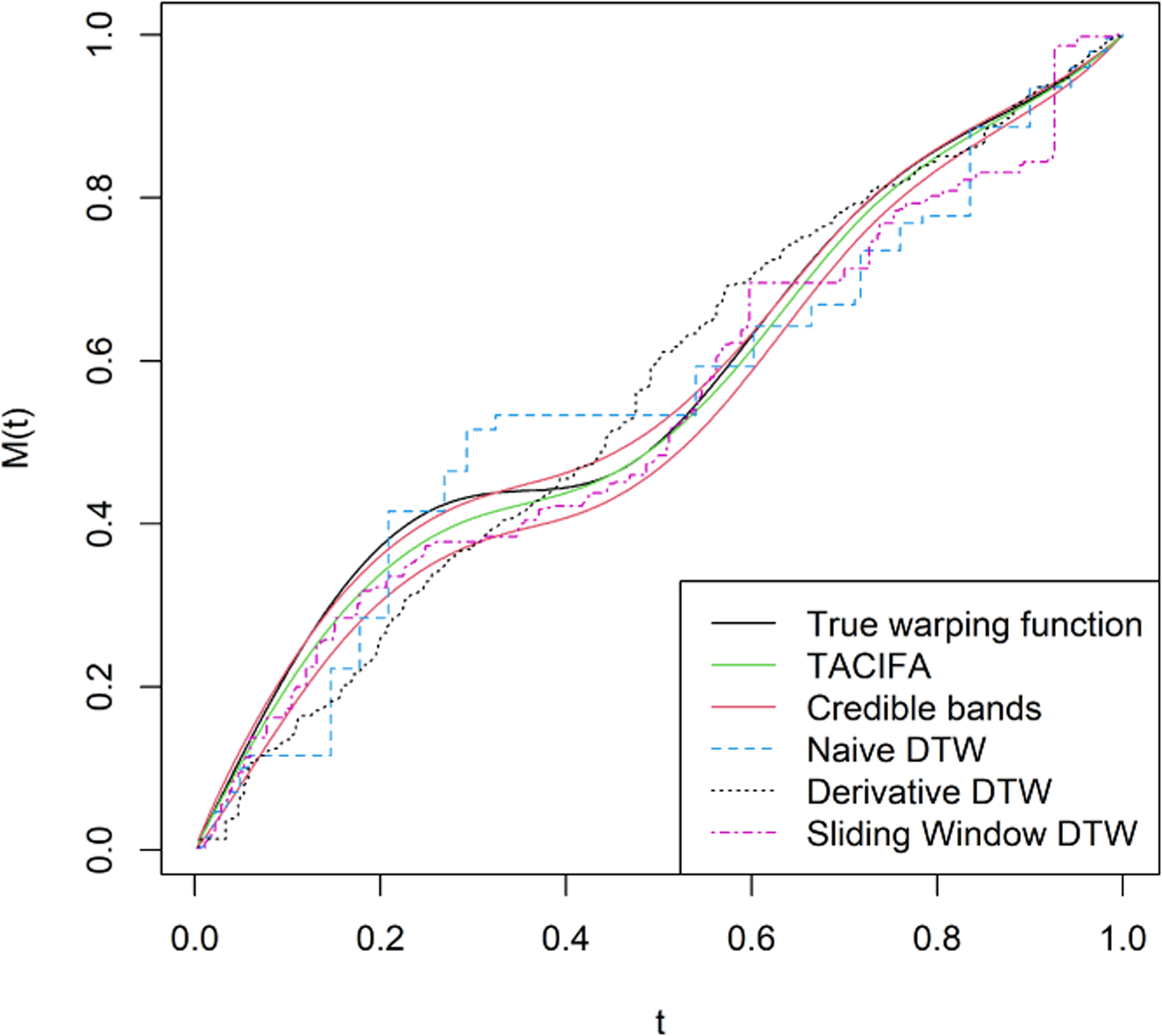
Estimated warping function for simulated data in a setting similar to Simulation Case 1, but with different true warping function. The black curve is the true warping function *M*_0_(*t*) = {(0.33 sin(2*πt*))^2^ + *t*^2^}^0.5^, the green curve is the estimated function, 95% credible bands are shown in red. Naive DTW and Sliding window DTW curves are indistinguishable. Of all the methods tested, the TACIFA estimated warping function is closest to the true warping function.

Figure 4 illustrates estimated shared loading matrices along with the true loading matrix. The estimated loading matrices roughly match with the true loading structure. The individual specific loadings, however, are not reliably distinguishable as they are constructed as (**I**_*p*_ − **Λ**(**Λ**^*T*^ **Λ**)^−1^**Λ**^*T*^)**Γ**_*i*_. Thus, we only present our results for the shared loading matrix. Figure 3, however, shows that the ranks of the individual specific loading matrices and the shared loading matrices are all roughly accurate using the proposed importance measures. Next, we evaluate the accuracy of our estimated warping function and accompanying uncertainty quantification. The estimated warping function in Figure 5 is for the training set. The estimate by TACIFA is clearly the best among all methods tested. In Table 1, we compare the prediction MSE results of our method with two-stage methods, and show that TACIFA has the best performance. Furthermore, Figure 6 illustrates estimated warping curves for a different true warping function *M*_0_(*t*) = {(0.33 sin(2*πt*))^2^ + *t*^2^}^0.5^ which incorporates change in direct of mimicry. The TACIFA based estimate is again the best among all the other competing methods.

Finally we measure the similarity of the simulated data using the measure described in Section 4.2. If *ζ*_1*k*_(*t*) = sin(*kt*) as above, the similarity is 0.95. To confirm that this measure is sensitive to the similarity between two time series, as intended, we change the first multivariate time series relative to the other multivariate time series by changing the first individual specific latent factors *ζ*_1*k*_(*t*) systematically, and recalculating the similarity. When *ζ*_1*k*_(*t*) = *kt*, similarity drops from 0.95 to 0.89. When *ζ*_1*k*_(*t*) = (*kt*)^2^, similarity further reduces to 0.79. The warping function estimated for each of these pairs of time series deteriorates as the two multivariate time series become more distinct as expected. Two stage methods do much worse in these cases (Figure 5 of the Supplementary Materials).

### 6.2 Simulation case 2

In Simulation Case 2, each series reflects a circle changing into an ellipse over time, similar to a mouth gaping and subsequently closing. The area of the shape is kept fixed by modifying the major and minor axis appropriately. The area of an ellipse, with *a* and *b* as the lengths of the major and minor axes, is given by *πab*. Thus to have the area remain fixed we need *ab*=constant. We maintain the constant to be 2. With the same true warping function *M*_0_(*t*) as in the previous simulation, the values for major and minor axes are linked over time across the two individuals. We let *ax*(*t*) = 2(*t* + 1) where *t*’s are 500 equidistant values between 1/500 and 1 and *bx*(*t*) = 2/(*t* + 1); here *ax*(*t*) and *bx*(*t*) are major and minor axes of the ellipse at time *t* corresponding to *X*_*t*_. At *t* = 0, it is a circle. For the second series we then have *ay*(*t*) = 2(*t*^0.5^ + 1) and *by*(*t*) = 2/(*t*^0.5^ + 1). We consider the pair of Cartesian coordinates of 12 equidistant points across the perimeter of the ellipse as features (yielding 24 features in total). The features correspond to 12 equidistant angles in [0, 2*π*). Let *θ*_1_, …, *θ*_12_ be those angles. Then *X*_*it*_ = (*ax*(*t*) sin(*θ*_*i*_), *bx*(*t*) cos(*θ*_*i*_)) and *Y*_*it*_ = (*ay*(*t*) sin(*θ*_*i*_), *by*(*t*) cos(*θ*_*i*_)).

The choices of hyperparameters and the number of MCMC iterations are all the same as in the previous simulation case. We again set *K*_1_ = *K*_2_ = *J* = *K* and fit the model for 4 different choices as before. The best choice based on the out of sample prediction for this case is *K* = 8. We have a pair of 24 dimensional time series. The X or Y coordinate is zero for the following four features *θ*_*i*_ = 0, *π* and *θ*_*i*_ = *π*/2, 3*π*/2. Thus, the warping should not have any effect on these features and should not contribute to the individual-specific space. The remaining 20 features represent 10 features and their mirror images with respect to either the major or minor axis. Thus, we might predict that the shared space should have 10 independent factors, which is consistent with the results displayed in Figure 6 of the Supplementary Materials. As there are 12 features, the individual-specific space should ideally have around two important factors. This is the case for one of the two individual-specific plots in Figure 6 of the Supplementary Materials. For the other individual, there is one more moderately important factor if we set a threshold of 0.9 on the importance measure SP. Figure 8 compares the estimates of the warping function when signal-to-noise ratio is low. Although our estimates perform much better than the rest, the width of credible bands expands with increasing error variance. Since the magnitudes of the features are very small, even noise with variance 1 or 1.5^2^ is large.

**Figure 7:**
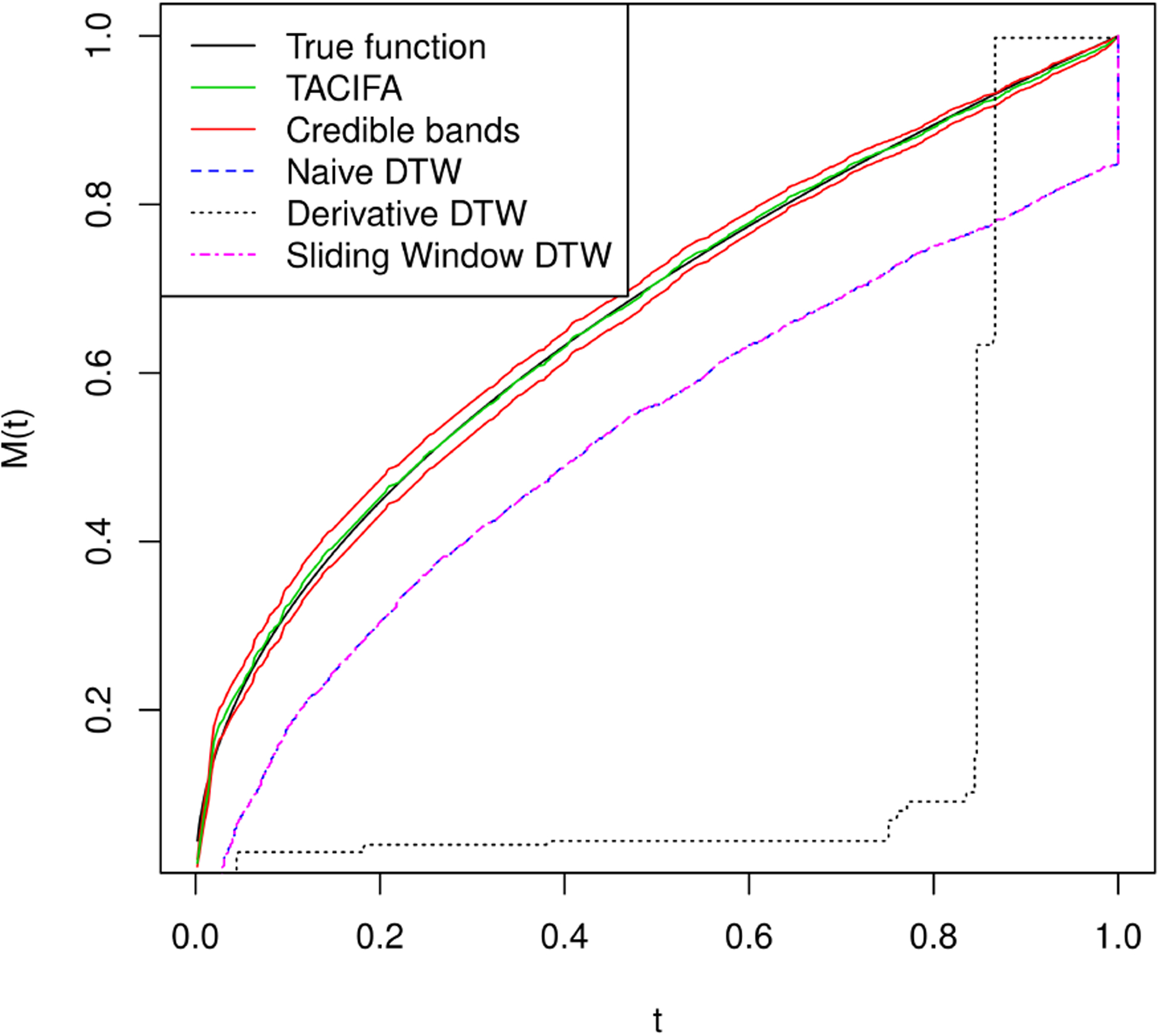
Estimated warping functions for Simulation case 2. The black curve is the true warping function *M*_0_(*t*) = *t*^0.5^. The green curve is the TACIFA estimated function, with the 95% credible bands shown in red. Naive DTW and Sliding window DTW curves are indistinguishable. Of all the methods tested, the TACIFA estimated warping function is closest to the true warping function.

**Figure 8:**
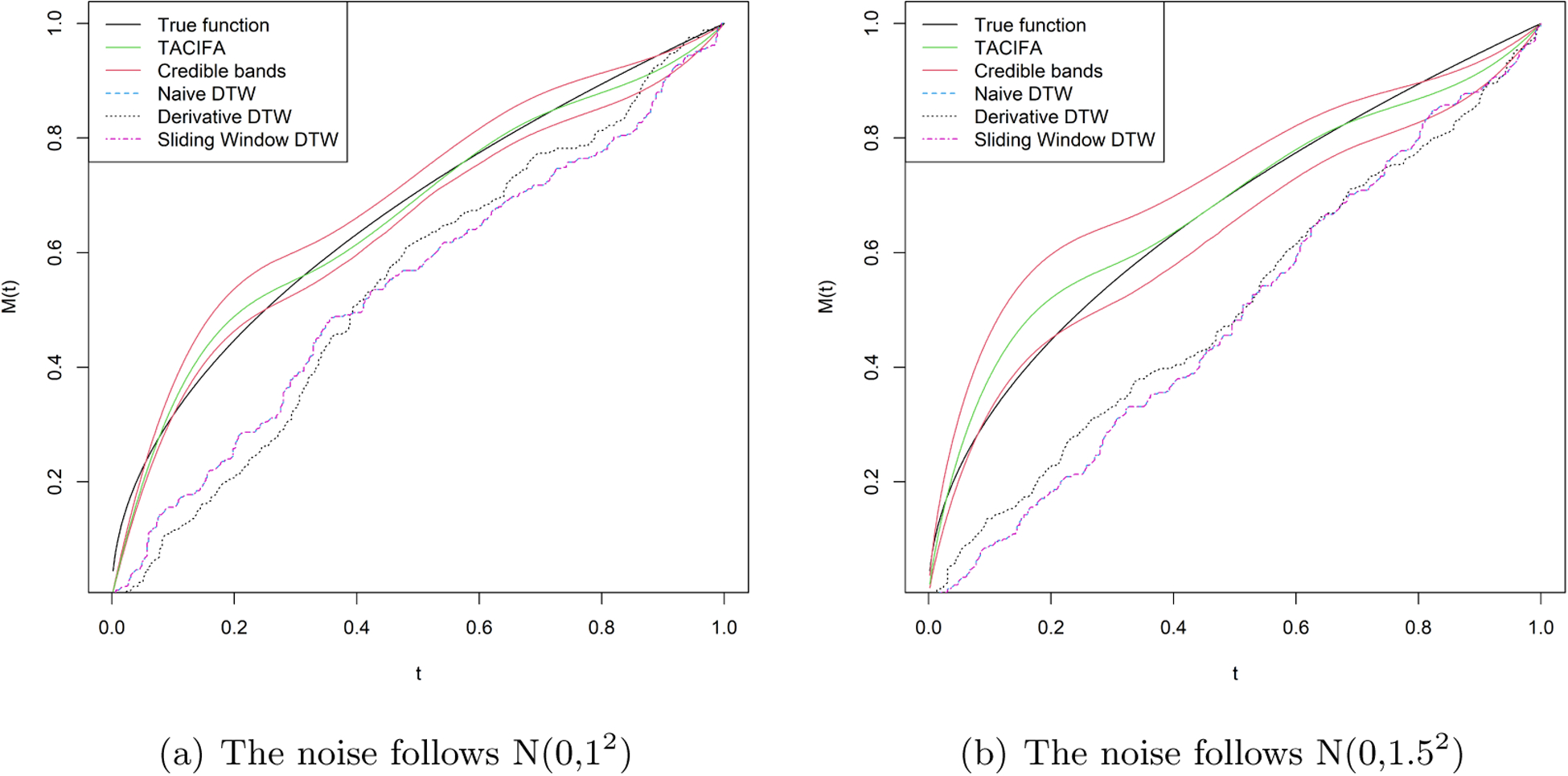
Estimated warping functions for Simulation case 2 with more noise added to the data.

**Figure 9:**
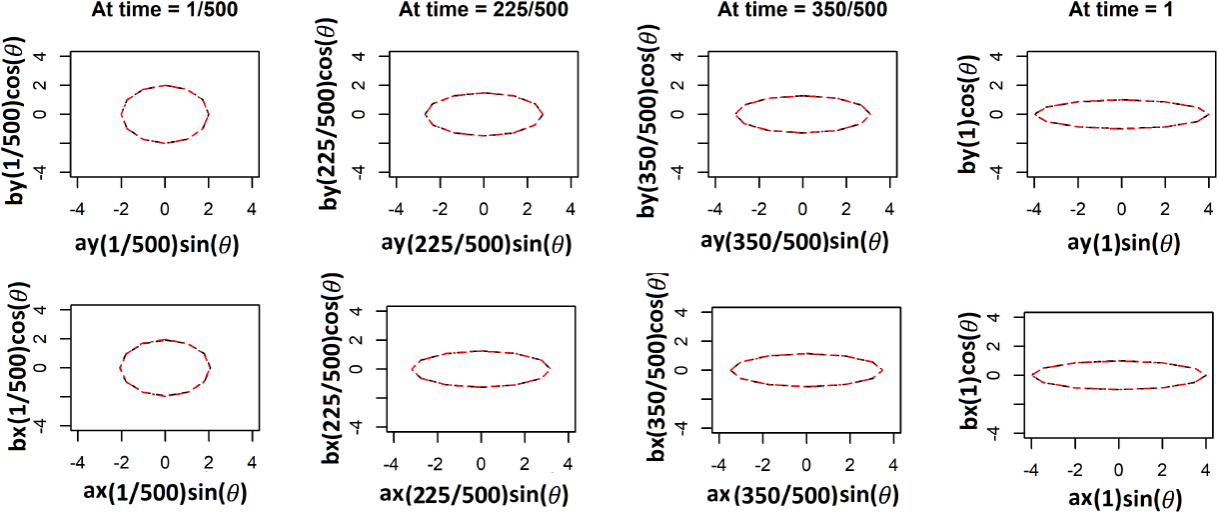
Results for simulation case 2. The first row corresponds to the co-ordinates (*ax*(*t*) sin(*θ*), *bx*(*t*) cos(*θ*)) for four choices of *t*, evaluated on a grid of *θ*. Likewise, the second row shows the co-ordinates of (*ay*(*t*) sin(*θ*), *by*(*t*) cos(*θ*))’s for the same choices of *t* and the *θ*-grid. Here *ax*(*t*) = 2(*t* + 1), *bx*(*t*) = 2/(*t* + 1) and *ay*(*t*) = 2(*t*^0.5^ + 1), *by*(*t*) = 2/(*t*^0.5^ + 1). The black dashed lines represent true curves at four time points and the red dashed lines are the estimated curves. The fit is excellent so that they almost lie on top of each other. At *t* = 1, *X* and *Y* both have the same shape.

We plot the estimated warping functions in Figure 7, and plot the estimated shapes in Figure 9. Figure 7 illustrates that the TACIFA-estimated warping function is once again the most accurate of the tested approaches. The TACIFA-estimated warping function is almost identical to the true curve, and has tightly concentrated credible bands. Figure 9 confirms that the TACIFA-estimated Cartesian coordinates of the 12 equidistant features are almost perfectly aligned with the true Cartesian coordinates. Quantifying these accuracies, we calculate the prediction TACIFA MSEs, which are 1.34 × 10^−6^ and 4.99 × 10^−6^ with 95% and 96% frequentist coverage within 95% posterior predictive credible bands for X and Y coordinates, respectively. In Table 2, we compare the results of our method with two-stage methods, and show that TACIFA again has the best performance, this time much more dramatically than in the first simulation. The method mtsdi gives similar prediction error to our method in the first simulation setup but fails to impute at any of the missing time points for the second simulation. MICE could impute in the first simulation, but only partially for the second simulation. Only missForest could produce results for both of the two simulations. Nonetheless, its prediction MSEs are much higher than those of our method.

## 7. Human Mimicry Application

We apply TACIFA to data from a simple social interaction in which one participant was instructed to imitate the head movements of another. The interaction occurred over Skype, and the videos of both participants were recorded. OpenFace software (Baltrusaitis et al., 2018) was used to extract regression scores for the X and Y coordinates of facial features around the mouth, as well as the pitch, yaw, and roll of head positions, from each frame of each video. These facial features are extracted and normalized before comparing the corresponding time series. Here, we analyze a session where one individual was instructed to imitate the other participant’s head movement throughout the interaction. We also apply our method to two related sessions where the role of imitator/imitate changes during the session, with results in Section 3 of Supplementary Materials. Although these social interactions were intentionally constrained to help assess the current methodology under consideration, they represent the types of dynamic social interactions that are of interest to psychologists, autism clinicians, and social robotics developers.

The duration of the experiment is rescaled into [0, 1]. The choices of hyperparameters for estimation are kept the same as in the two simulation setups above except for the number of B-splines. We again run a similar cross validation procedure, and set the number of bases at 8. We collect 5000 MCMC samples after 5000 burn-in samples. We truncate the columns of the loading matrices that have mean absolute contribution less than 0.0001. We plot the estimated warping function along with credible bands and the values of *SP*(**ΨΓ**_1_), *SP*(**ΨΓ**_2_), *SP*(**ΛΞ**_1_), and *SP*(**ΛΞ**_2_) as in the simulation analyses. Recall that *SP*_*i*,*j*_(*A*) = (|0.5 − *P* (*A*[*i*, *j*] > 0)|) /0.5 where *P* (*A*[*i*, *j*] > 0) stands for the posterior probability estimated from the MCMC samples of *A* after performing the post-processing steps defined in Section 4.1.

**Figure 10:**
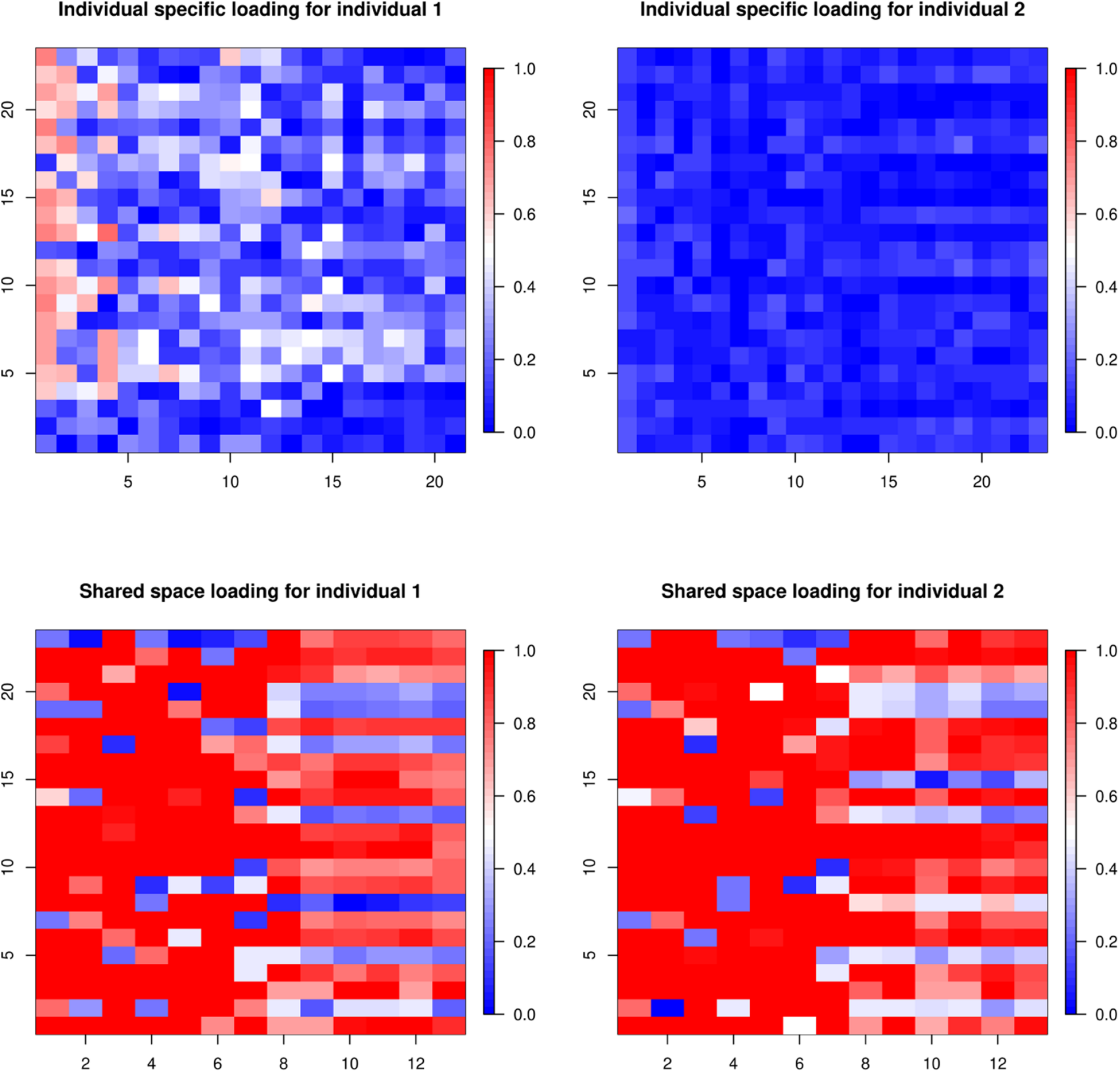
Plot of the summary measure as evidence of importance of the entries of loading matrices in human mimicry dataset (A). Each column represents one factor. The columns with higher proportion of red correspond to the factors with higher importance.

We apply TACIFA to the time courses of 20 facial features from around the mouth and chin, along with three predictors of head position. We begin by evaluating the loading matrices of the shared and individual factors. There should be a large shared space in this experiment, as we know one person was imitating the head movements of the other, and all of the features examined were related to the head. We plot *SP*(**ΨΓ**_1_), *SP*(**ΨΓ**_2_), *SP*(**Λ**), and *SP*(**ΛΞ**_2_) in Figure 10. Half of the 20 facial features examined in this experiment were roughly the mirror image of the others, due to facial symmetry. As a consequence, we might predict that the shared space should not have more than 13 factors. Consistent with this hypothesis, there are 13 important shared features in Figure 7. In addition, all of the features examined in this experiment are related to head movement, so we might predict very little individual variation in the time courses. This prediction is consistent with the low importance of all the individual-specific factors shown in Figure 10.

**Figure 11:**
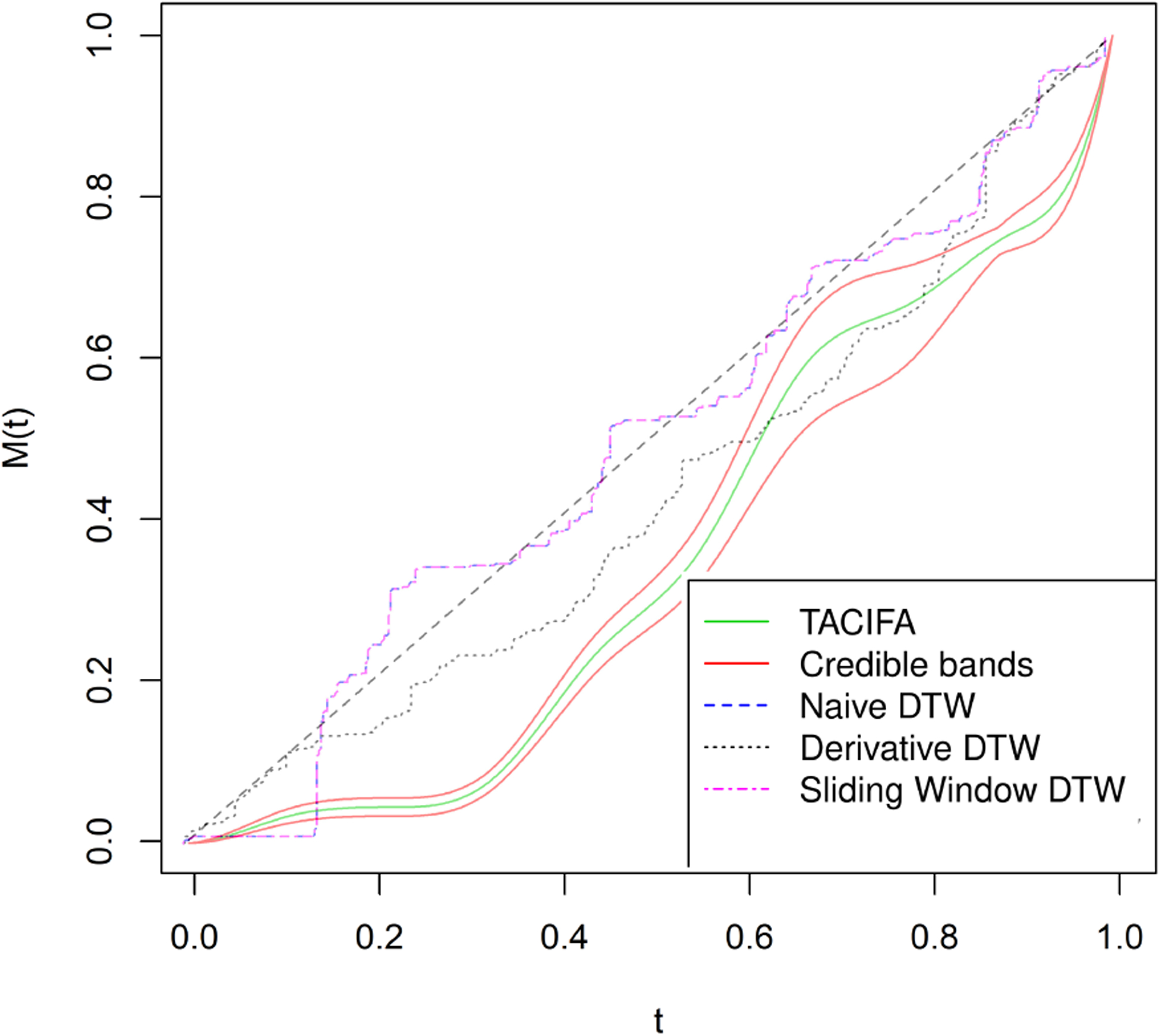
Estimated warping function in human mimicry dataset (A). The green curve is the estimated function along with the 95% pointwise credible bands in red. The estimated curve is always below the dashed line, indicating the second person is mimicked throughout the experiment

Next, we examine the TACIFA estimated warping function and accompanying uncertainty quantification. Figure 11 shows that the estimated warping function is below the *M*(*t*) = *t* line throughout the experiment. This indicates that the TACIFA approach correctly estimated that one individual was following the other individual in time through the experiment. Derivative DTW was the only other method that achieved that. Furthermore, all these methods also suggest that the participants switched leadership roles multiple times, which is not true.

Next, we compare the TACIFA out of sample prediction MSEs to those of two-stage approaches, and compute the similarity. The TACIFA MSEs are 4.25 and 2.21, with 95% and 98% frequentist coverage within 95% posterior predictive credible bands, relative to the estimated variances 4.34 and 2.61 for the first and second individuals, respectively. These MSEs are lower than those of the two stage approaches, which are around 9. A detailed table is in the Supplementary Materials.

Finally, we assess the similarity of the two time series and test whether greater numbers of features influence the similarity measure. Let **X**_*m*_ and **Y**_*m*_ denote the paired time series with *m* set of features (maximum of 10) around the chin along with the three predictors on head position. We have a total of 10 possible features in this analysis. We get Syn(**X**_3_, **Y**_3_)=0.80, Syn(**X**_6_, **Y**_6_)=0.85 and Syn(**X**_10_, **Y**_10_)=0.85. These high values are reasonable, since all the features examined will be influenced by head movement and head movements were intentionally coordinated. The results also indicate that similarity values increase as the number of relevant features increases.

## 8. Discussion

There are many possibilities of future research building on TACIFA. It is natural to generalize to *D* many matrices, which would require *D* different individual-specific loadings **Γ**_1_, …, **Γ**_*D*_ along with *D* − 1 different warping functions. In addition, in settings such as our motivating social mimicry application, there may be data available from *n* pairs of interacting individuals. In such a case, it is natural to develop a hierarchical extension of the proposed approach that can borrow information across individuals and make inferences about population parameters. Another direction is to build static Bayesian models to estimate the joint and individual structures under the orthogonality assumption by dropping the warping function from our proposed model to accounting for group differences. The current implementation for updating **Λ** prohibits its use for large *p* as the computational complexity in updating a *p* × *r* dimensional **Λ** at each iteration is of order *rp*^2^. Thus, developing computationally efficient posterior computation algorithms is another direction to ensure broader applicability of our proposed method. Future work will also consider the cases where the data matrices **X** and **Y** have an unequal number of time points. Although theoretically our proposed model can accommodate this case, the computational complexity may be high.

A further important and challenging direction is to generalize the proposed methods to allow for more complex types of interactions. Two individuals who are interacting may not simply imitate each other, but have more nuanced and diverse types of coordination. For example, one individual may nod their head or laugh in response to the funny facial expressions another individual intentionally makes, or one individual may close their eyes when the other individual sticks out their tongue. Accommodating such complexity will require a more complex dynamic latent structure than that described here.

## Supplementary Material

Supple text

## Figures and Tables

**Table 1:** Prediction MSEs of the first and second time series in Simulation 1. using two-stage methods. The top row indicates the R package used to impute, and the first column indicates the warping method. The two-stage prediction MSEs are all greater than the TACIFA prediction MSEs (1.01, 1.02).

	missForest	MICE	mtsdi
Naive DTW	(6.12, 9.66)	(8.65,9.70)	(1.03,1.03)
Derivative DTW	(6.37, 9.49)	(8.06,9.80)	(1.03,1.03)
Sliding DTW	(7.15, 10.55)	(9.61,10.39)	(1.03,1.03)

**Table 2:** Prediction MSEs of the first and second time series in Simulation 2 using two-stage methods. The top row indicates the R package used to impute, and the first column indicates the method used to warp. mtsdi could not impute at any of the testing time points in this simulation. The two-stage prediction MSEs are all greater than the TACIFA prediction MSEs (1.34 × 10^−6^, 4.99 × 10^−6^).

	missForest	MICE	mtsdi
Naive DTW	(0.12,0.07)	(0.18,0.09)	(−,−)
Derivative DTW	(0.12,0.07)	(0.15,0.07)	(−,−)
Sliding DTW	(0.12,0.07)	(0.14,0.05)	(−,−)
