# Supplementary material for: Bayesian time-aligned factor analysis of paired multivariate time series
Source: J Mach Learn Res. Author manuscript; Available in PMC 2022 Jun 23. (PMC9221555)
Supplement: Supple text [file NIHMS1815557-supplement-Supple_text.pdf]

Journal of Machine Learning Research 1 (2019) – Submitted 5/19; Published –/–

Supplementary materials for "Bayesian time-aligned factor analysis of paired multivariate time series"

Arkaprava Roy ark007@ufl.edu
Department of Biostatistics
University of Florida
Durham, NC 27708-0251, USA

Jana Schaich-Borg js524@duke.edu
Social Science Research Institute
Duke University
Durham, NC 27708-0251, USA

David B Dunson dunson@duke.edu
Department of Statistics
Duke University
Durham, NC 27708-0251, USA

Editor:

1. Cubic B-splines

Consider knot-points $t_{-2} = t_{-1} = t_0 = t_1 = A < t_2 < \cdots < B = t_K = t_{K+1} = t_{K+2} = t_{K+3}$, where $t_{1:K}$ are equidistant with $\delta = (t_2 - t_1)$. For $j = 1, 2, \ldots, (K+3)$, cubic B-splines $b_{3,j}$ are defined as

$$b_{3,j}(x) = \begin{cases} \frac{1}{6\delta^3}\{(x - t_{j-2})\}^3 & \text{if } t_{j-2} \leq x < t_{j-1}, \\ \frac{1}{6\delta^3}\{\delta^3 + 3(x - t_{j-1})\delta^2 + 3(x - t_{j-1})^2\delta - 3(x - t_{j-1})^3\} & \text{if } t_{j-1} \leq x < t_j, \\ \frac{1}{6\delta^3}\{\delta^3 + 3(t_{j+1} - x)\delta^2 + 3(t_{j+1} - x)^2\delta - 3(t_{j+1} - x)^3\} & \text{if } t_j \leq x < t_{j+1}, \\ \frac{1}{6\delta^3}\{(t_{j+2} - x)\}^3 & \text{if } t_{j+1} \leq x < t_{j+2}, \\ 0 & \text{otherwise.} \end{cases}$$

2. Proofs of the theorems

In this section, the detailed proofs of the theorems are presented.

2.1 Proof of Theorem 1

We can write $\|\Lambda_1^{(1)} - \Lambda_1^{(2)} R_1\|_F^2 = trace(\Lambda_1^{(1)} - \Lambda_1^{(2)} R_1)^T(\Lambda_1^{(1)} - \Lambda_1^{(2)} R_1) = trace\big[(\Lambda_1^{(1)})^T\Lambda_1^{(1)}) + \Lambda_1^{(2)})^T\Lambda_1^{(2)}) - 2(\Lambda_1^{(1)})^T\Lambda_1^{(2)} R_1\big]$, using the properties of trace of a matrix.

©2019 .

License: CC-BY 4.0, see https://creativecommons.org/licenses/by/4.0/. Attribution requirements are provided at http://jmlr.org/papers/v1/ark007@ufl.edu.html.

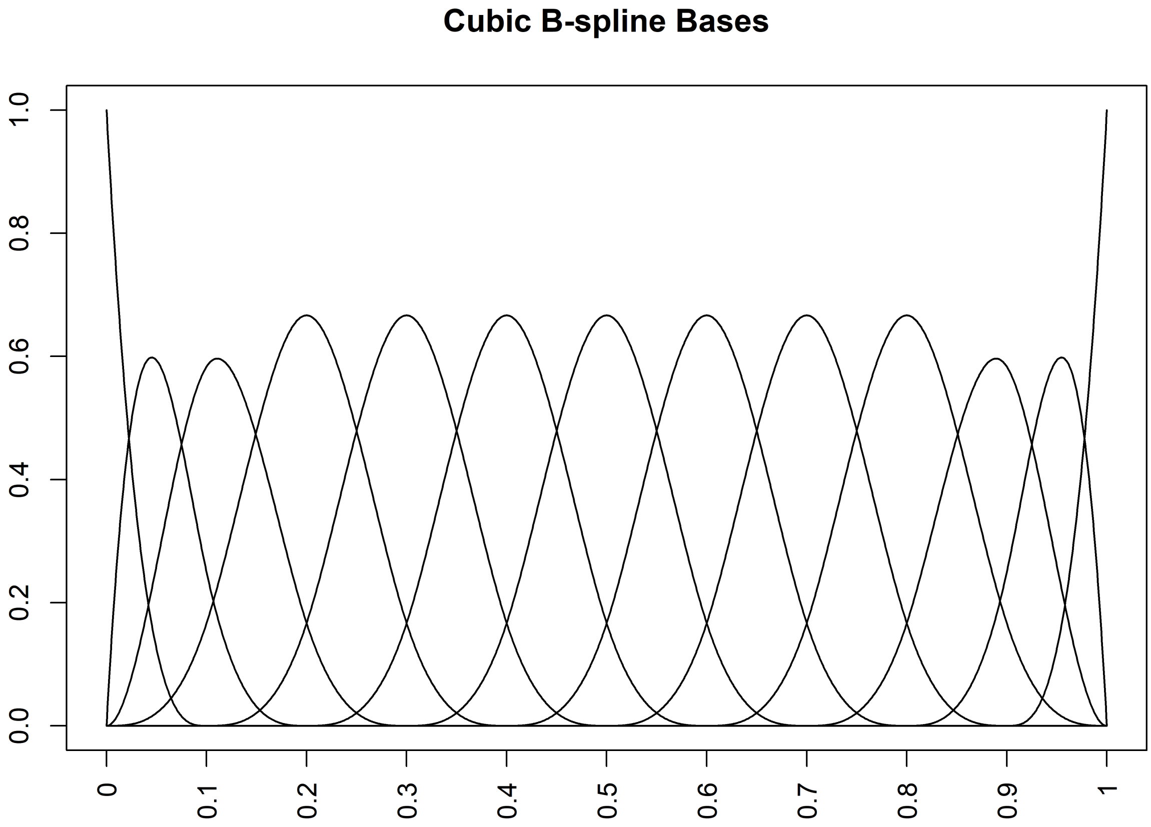

Figure 1: Plot of 13 cubic B-splines on $[0, 1]$ defined using 10 knot points that divide $[0, 1]$ into $K = 10$ equal sub-intervals.

Thus the minimization problem $\|\Lambda_1^{(1)} - \Lambda_1^{(2)} R_1\|_F^2$ is equivalent to the maximization of $trace((\Lambda_1^{(1)})^T \Lambda_1^{(2)} R_1) = trace(Q_1 D Q_2^T R_1) = \sum_{i=1}^r D_{ii} \sum_{j=1}^r x_{ij} y_{ij}$, where x_{ij} and y_{ij} are the (i, j)-th entries of Q_1 and $(Q_2^T R_1)^T$. The SVD of $(\Lambda_1^{(1)})^T \Lambda_1^{(2)}$ is $Q_1 D Q_2^T$ with Q_1 and Q_2 orthonormal matrices. Since Q_1 and $(Q_2^T R_1)^T$ are orthonormal matrices, the above sum is maximized when $x_{ij} = y_{ij}$ for all (i, j) by the Cauchy-Schwarz inequality. Thus $Q_1 = (Q_2^T R_1)^T$ which implies $R_1 = Q_2 Q_1^T$.

2.2 Proof of Theorem 2

We consider $\Lambda_1 = \Lambda \Xi_1$ and $\Lambda_2 = \Lambda \Xi_2$. Due to orthogonality between individual-specific and shared space loading matrices, the decomposition into shared space and individual-specific means is identifiable. Let us consider $\Lambda_{11}\eta_1(t) = \Lambda_{12}\eta_2(t)$ and $\Lambda_{21}\eta_1(M_1(t)) = \Lambda_{22}\eta_2(M_2(t))$. We consider M_1 and M_2 to be strictly increasing

functions. We assume M_1 and M_2 are different. By simple arguments we show that this is a contradiction.

For two full-rank matrices P and Q, we have $\Lambda_{11} = \Lambda_{12}P$, $\eta_1(t) = P^{-1}\eta_2(t)$ and $\Lambda_{21} = \Lambda_{22}Q$, $\eta_1(M_1(t)) = Q^{-1}\eta_2(M_2(t))$. This implies

$$\begin{aligned} Q^{-1}\eta_2(M_2(t)) &= P^{-1}\eta_2(M_1(t)), \\ \eta_2(M_2(t)) &= QP^{-1}\eta_2(M_1(t)). \end{aligned} \tag{1}$$

The rank is r. The two functions M_1 and M_2 are increasing, thus they are invertible. This gives us $\eta_2(t) = QP^{-1}\eta_2(M_1 \circ M_2^{-1}(t))$. Let $f(t) = M_1 \circ M_2^{-1}(t)$ and f^k be k many self convolutions of f. Then we have

$$\eta_2(t) = (QP^{-1})^k \eta_2(f^k(t)),$$

for all positive integers k. Such a result also holds for the inverse of f, $f^{-1}()$. By construction, f and f^{-1} are monotone. If $f(t) \leq t$, $f^k(t)$ is a decreasing sequence in k for the given t. Then $f^{-1}(t) > t$ and the set of its self convolutions is an increasing sequence in k. Then we have using product rule limit,

$$\eta_2(t) = \lim_{k\to\infty} (QP^{-1})^k \lim_{k\to\infty} \eta_2(f^k(t)) = B\eta_2(0),$$

where $\lim_{k\to\infty}(QP^{-1})^k = B$. Due to boundedness of η_2, product rule is possible as both of the two limits exist. Similarly we have $\eta_2(t) = B^{-1}\eta_2(T)$. If $\eta_2(0) = 0$ and $\eta_2(T) = 0$, then $\eta_2(t) = 0$ for all t. This is not possible. Also we have $\eta_2(T) = B^2\eta_2(0)$. Thus we must have $\eta_2(0) \neq 0$ and $\eta_2(T) \neq 0$. We then have $M_1(T) = 1 = M_2(T)$ and $M_1(0) = 0 = M_2(0)$. Thus for all t such that $f(t) \leq t$, $\eta_2(t) = B\eta_2(0)$. Similarly for $f(t) \geq t$, $\eta_2(t) = B\eta_2(T) = B^3\eta_2(0)$. For continuity, we must have $B = I$. This is because we have $B^2\eta_2(0) = \eta_2(0)$ which automatically implies $B^2\eta_2(t) = \eta_2(t)$ for all t, implying $B = I$.

Hence, it implies again that $\eta_2(t)$ is constant over t. Same result holds for η_1 as well. This is a contradiction as the condition is that $\eta(t)$ is not constant over t. Thus the only possibility we have is $M_1 = M_2$.

2.3 Proof of Theorem 3

We apply the posterior concentration result from the Section 8.3 of Ghosal and Van der Vaart (2017) for non identically distributed observations. It will require verifying the prior concentration in an ϵ_n^2 neighborhood around the truth, existence of exponentially consistent tests for the truth against an alternative in terms of the metric d_n with at most error probabilities $\exp(-c_1 n\epsilon_n^2)$, and a "sieve" in the parameter space with at least $1 - \exp^{-c_2 n\epsilon_n^2}$ probability that can be covered by at most $\exp(c_3 n\epsilon_n^2)$ balls of radius ϵ_n for some constants c_1, c_2 and c_3 such that $c_2 > c_1 + 4$. In our model we have $0 \leq t_i \leq 1$ for all $1 \leq i \leq n$.

For $q, q^* \in$ the space of probability measure $\mathcal{P}$, let

$$K(q^*, q) = \int q^* \log \frac{q^*}{q} \qquad V(q^*, q) = \int q^* \log^2 \frac{q^*}{q}.$$

We consider the paired data (X_i, Y_i) as the *i-th* data point. For $\mu_i^* = (\zeta_1^*(t_i) + \eta^*(t_i), \zeta_2^*(t_i) + \eta^*(M^*(t_1))), \Sigma^* = (\Sigma_1^*, \Sigma_2^*)$ $q_i^* = \mathrm{MVN}(\mu_i^*, \Sigma^*)$ and $q_i = \mathrm{MVN}(\mu_i, \Sigma)$, by simple calculations

$$K(q_i^*, q_i) = \sum_{j=1}^{2}\sum_{i=1}^{p} \log\left(\frac{\sigma_{ji}}{\sigma_{ji}^*}\right) - \frac{1}{2}\left[p - \sum_{i=1}^{p}\frac{(\mu_{ij}^* - \mu_{ij})^2}{\sigma_{ji}^2} - \sum_{i=1}^{p}\frac{\sigma_{ji}^{*2}}{\sigma_{ji}^2}\right],$$
$$V(q_i^*, q_i) = \sum_{j=1}^{2}\sum_{i=1}^{p}\frac{1}{2}\left(\frac{\sigma_{jj}^{*2}}{\sigma_{ji}^2} - 1\right)^2 + \sum_{i=1}^{p}\frac{(\mu_{ij}^* - \mu_{ij})^2}{\sigma_{ji}^4}\sigma_{ji}^{*2}.$$

We denote $\mu = (\mu_1, \ldots, \mu_n)$ as all the means stacked together. Let μ_0 and σ_0 stand for the truth of μ and σ. Let μ^* be such that $\|\mu_0 - \mu^*\| > \epsilon_n$. By Lemma 8.27 of Ghosal and Van der Vaart (2017) for bounded σ one can construct an exponentially consistent test for (μ_0, σ_0) against $(\mu_1, \sigma_1 : \|\mu_1 - \mu^*\| < \epsilon_n/2)$. Using negative log affinity measure one can construct a test with unbounded support as in Ning et al. (2020). To keep the proof simple, we consider the additional boundedness assumption.

$$\begin{aligned} &d^2((\zeta_{11}, \zeta_{21}, \eta_1, M_1), (\zeta_{12}, \zeta_{22}, \eta_2, M_2)) \\ &\quad\lesssim n^{-1}\sum_{i=1}^{n}\left[\|\zeta_{11}(t_i) - \zeta_{12}(t_i)\|_2^2 + \|\zeta_{21}(t_i) - \zeta_{22}(t_i)\|_2^2 + \|\eta_1(t_i) - \eta_2(t_i)\|_2^2\right. \\ &\qquad\left. + \|\eta_1(M_1(t_i)) - \eta_2(M_2(t_i))\|_2^2\right] \end{aligned}$$

where $\lesssim$ stands for inequality up to a constant multiple. Thus to bound ϵ_n-metric entropies, the logarithm of the number of ϵ_n-balls needed to cover a set, we can consider the functions η and M separately.

To proceed with the posterior concentration result we consider a sieve in the parameter space of the form, $\mathcal{G}_n = \{\beta_1, \beta_2, \beta, K, J, \sigma : \|\beta_1\|_\infty \le B_{1n}, \|\beta_2\|_\infty \le B_{2n}, \|\beta\|_\infty \le B_n, K \le K_n, J \le J_n, 1/n \le \sigma_{ij} \le e^{c' n\epsilon_n^2}, j = 1, \ldots, p, i = 1, 2\}$. We have for two sets of parameters $\Theta = (\zeta_1, \zeta_2, \eta, M, \sigma)$ and $\Theta^* = (\zeta_1^*, \zeta_2^*, \eta^*, M^*, \sigma^*)$,

$$n^{-1}\|\eta(M(t_i)) - \eta^*(M^*(t_i))\|_2^2 \lesssim B_n^2 \max_l |\gamma_l - \gamma_l^*|^2,$$

and

$$n^{-1}\|\eta(t_i) - \eta^*(t_i)\|_2^2 \lesssim \max_l |\beta_l - \beta_l^*|^2.$$

The error in approximating the function η can be uniformly bounded in an order of $\bar{K}_n^{-\iota}$ using K_n B-spline basis functions. Similarly for ζ_1 and ζ_2 these are up

to order $\bar{K}_{1n}^{-\iota_1}$ and $\bar{K}_{2n}^{-\iota_2}$. Also for M with $\bar{J}_n$, it is $\bar{J}_n^{-\iota'}$. Thus we have $\epsilon_n \geq \max(\bar{K}_{1n}^{-\iota_1}, \bar{K}_{2n}^{-\iota_2}, \bar{K}_n^{-\iota}, \bar{J}_n^{-\iota'})$. For our prior $\Pi(\max_l |\gamma_l - \gamma_l^*| \leq c\epsilon_n) > \epsilon^{\bar{J}_n}$, $\Pi(\max_l |\beta_{1l} - \beta_{1l}^*| \leq c\epsilon_n) > \epsilon^{\bar{K}_{1n}}$,$\Pi(\max_l |\beta_{2l} - \beta_{2l}^*| \leq c\epsilon_n) > \epsilon^{\bar{K}_{2n}}$, and $\Pi(\max_l |\beta_l - \beta_l^*| \leq c\epsilon_n) > \epsilon^{\bar{K}_n}$. The contraction rate ϵ_n must be worse than parametric rate n^{-1}. Thus we have $\epsilon^{\bar{K}_{1n}+\bar{K}_{2n}+\bar{K}_n+\bar{J}_n} > \exp(-c'(\bar{K}_{1n} + \bar{K}_{2n} + \bar{K}_n + \bar{J}_n)\log n)$ for some $c' > 0$. For a pre-rate $\bar{\epsilon}_n$ we have

$$(\bar{K}_{1n}^{-\iota_1} + \bar{K}_{2n}^{-\iota_2} + \bar{K}_n^{-\iota} + \bar{J}_n^{-\iota'}) \lesssim \bar{\epsilon}_n, \quad (\bar{K}_{1n} + \bar{K}_{2n} + \bar{K}_n + \bar{J}_n)\log n \lesssim n\bar{\epsilon}_n^2. \tag{2}$$

The actual contraction might be little higher than this pre-rate. It depends on the prior. The bound for ϵ_n-entropy of the sieve will be a constant multiple of $(K_{1n} + K_{2n} + K_n + J_n)\log n + n\epsilon_n^2$. Taking B_{1n}, B_{2n}, B_n as a polynomial of n, to satisfy the conditions on the sieve according to the general theory, we need

$$\begin{aligned} K_{1n}(\log n)^{b_{13}} + K_{2n}(\log n)^{b_{23}} + K_n(\log n)^{b_3} + J_n(\log n)^{b_3'} &\gtrsim n\bar{\epsilon}_n^2, \\ (K_{1n} + K_{2n} + K_n + J_n)e^{-bn^2} &\lesssim \exp[-n\bar{\epsilon}_n^2]. \end{aligned} \tag{3}$$

If we consider $\bar{K}_{in} \asymp (n/\log n)^{1/(2\iota_i+1)}$, for $i = 1, 2$, $\bar{K}_n \asymp (n/\log n)^{1/(2\iota+1)}$, $\bar{J}_n \asymp (n/\log n)^{1/(2\iota'+1)}$ in (2), this leads to $\bar{\epsilon}_n \asymp (n/\log n)^{-\bar{\iota}/(2\bar{\iota}+1)}$ where $\bar{\iota} = \min\{\iota, \iota_1, \iota_2, \iota'\}$. Now to satisfy (3), we need

$$K_{in} \asymp n^{1/(2\iota_i+1)}(\log n)^{\iota_i/(2\iota_i+1)+(1-b_{i3})/2},$$

for $i = 1, 2$,

$$K_n \asymp n^{1/(2\iota+1)}(\log n)^{\iota/(2\iota+1)+(1-b_3)/2}, \quad J_n \asymp n^{1/(2\iota'+1)}(\log n)^{\iota'/(2\iota'+1)+1-b_3}.$$

Thus final rate ϵ_n becomes,

$$n^{-\bar{\iota}/(2\bar{\iota}+1)}(\log n)^{\bar{\iota}/(2\bar{\iota}+1)+(1-\bar{b}_3)/2},$$

where $\bar{\iota} = \min\{\iota, \iota_1, \iota_2, \iota'\}$ and $\bar{b}_3 = \min\{b_3, b_3', b_{13}, b_{23}\}$.

2.3.1 Proof of Corollary 1

The empirical ℓ_2-distance is given by,

$$\begin{aligned} &d_1^2((\Psi_1, \Lambda_1, \Gamma_{11}, \Gamma_{12}, \Xi_{11}, \Xi_{12}, \zeta_{11}, \zeta_{21}, \eta_1, M_1), (\Psi_2, \Lambda_2, \Gamma_{21}, \Gamma_{22}, \Xi_{21}, \Xi_{22}, \zeta_{12}, \zeta_{22}, \eta_2, M_2)) \\ &\quad = \frac{1}{n}\sum_{i=1}^n \big[\|\Psi_1\Gamma_{11}\zeta_{11}(t_i) - \Psi_2\Gamma_{12}\zeta_{12}(t_i)\|_2^2 + \|\Psi_1\Gamma_{21}\zeta_{21}(t_i) - \Psi_2\Gamma_{22}\zeta_{22}(t_i)\|_2^2 \\ &\qquad + \|\Lambda_1\Xi_{11}\eta_1(t_i) - \Lambda_2\Xi_{12}\eta_2(t_i)\|_2^2 + \|\Lambda_1\Xi_{21}\eta_1(M_1(t_i)) - \Lambda_2\Xi_{22}\eta_2(M_2(t_i))\|_2^2\big]. \end{aligned}$$

For the original model, test constructions will remain the same. We only need to verify Kullback-Leibler prior positivity. We can show that

$$\|\Psi_1\Gamma_{i1}\zeta_{i1} - \Psi_0\Gamma_{i0}\zeta_{i0}\|_2 \leq \|\Psi_1 - \Psi_0\|_2 + \|\Gamma_{i1} - \Gamma_{i0}(t)\|_2 + \|\zeta_{i1} - \zeta_{i0}\|_\infty,$$

for $i = 1, 2$ and

$$\|\Lambda_1 \Xi_{11} \eta_1 - \Lambda_0 \Xi_{10} \eta_0\|_2 + \|\Lambda_1 \Xi_{21} \eta_1(M_1) - \Lambda_0 \Xi_{20} \eta_0(M_0)\|_2 \leq \\ \|\Lambda_1 - \Lambda_0\|_2 + \|\eta_1 - \eta_0\|_\infty + \|M_1 - M_0\|_\infty + \|\Xi_{11} - \Xi_{10}\|_2 + \|\Xi_{21} - \Xi_{20}\|_2.$$

If the latent factors and loading matrices are close to their true values, then the means of individual-specific and shared space means are close to the corresponding true means. We have already proved that these means converge to their true values in Theorem 3. Thus the Kullback-Leibler divergence converges to zero. This completes the proof of the Corollary.

3. Posterior update of Γ_1, Γ_2, Ξ_1, Ξ_2 and β_1, β_2, β

To update Γ_1: The posterior mean and variance for $vec(\Gamma_1)$ are given below. Here $vec(\Gamma_1)$ is the vectorized version of the matrix Γ_1. Let us define a time varying matrix $T(t)$ of dimension $p \times pr_1$ and *i-th* row of $T(t)$ is $T_i(t) = \Psi_i * \zeta_1(t)$, where $*$ denotes convolution of *i-th* row of Ψ, and $\zeta_1(t)$. Let $V_{pm}^{\Gamma_1}$ and $M_{pm}^{\Gamma_1}$ denote the posterior variance and mean respectively. Then,

$$V_{pm}^{\Gamma_1} = \Big(\sum_i T_i(t)^T \Sigma_1^{-1} T_i(t) + \text{diagonal}(vec(PV)) \Big)^{-1},$$

where the matrix $PV_{p \times r_1}$ is defined as $PV_{lk} = \Phi_{11,lk} \tau_{11,k}$

$$M_{pm}^{\Gamma_1} = V_{pm} \sum_i T_i(t)^T (X_{it} - \Lambda_i \Xi_1 \eta(t))$$

To update Γ_2: The posterior mean and variance for $vec(\Gamma_2)$ are given below. Here $vec(\Gamma_2)$ is the vectorized version of the matrix Γ_2. Define a time varying matrix $T(t)$ of dimension $p \times pr_2$ with the *i-th* row of $T(t)$ being $T_i(t) = \Psi_i * \zeta_2(t)$. Let $V_{pm}^{\Gamma_2}$ and $M_{pm}^{\Gamma_2}$ be the posterior variance and mean respectively. Then,

$$V_{pm}^{\Gamma_2} = \Big(\sum_i T_i(t)^T \Sigma_2^{-1} T_i(t) + \text{diagonal}(vec(PV)) \Big)^{-1},$$

where the matrix $PV_{p \times r_2}$ is defined as $PV_{lk} = \Phi_{12,lk} \tau_{12,k}$

$$M_{pm}^{\Gamma_2} = V_{pm} \sum_i T_i(t)^T (Y_{it} - \Lambda_i \Xi_2 \eta(M(t)))$$

To update Ξ_1: The posterior mean and variance for $vec(\Xi_1)$ are given below. Here $vec(\Xi_1)$ is the vectorized version of the matrix Ξ_1. Define a time varying matrix $T(t)$ of dimension $p \times pr$ with the *i-th* row of $T(t)$ as $T_i(t) = \Lambda_i * \eta(t)$. Let $V_{pm}^{\Xi_1}$ and $M_{pm}^{\Xi_1}$ be the posterior variance and mean respectively. Then,

$$V_{pm}^{\Xi_1} = \Big(\sum_i T_i(t)^T \Sigma_1^{-1} T_i(t) + \text{diagonal}(\omega_r) \Big)^{-1},$$

where ω_r is vector with ω replicated r times, and

$$M_{pm}^{\Xi_1} = V_{pm} \sum_i T_i(t)^T (X_{it} - \Psi_i \Gamma_1 \zeta_1(t)).$$

To update Ξ_2: The posterior mean and variance for $vec(\Xi_2)$ are given below. Here $vec(\Xi_2)$ is the vectorized version of the matrix Ξ_2. Let us define a time varying matrix $T(t)$ of dimension $p \times pr$ with the *i-th* row $T_i(t) = \Lambda_i * \eta(M(t))$. Let $V_{pm}^{\Xi_2}$ and $M_{pm}^{\Xi_2}$ be the posterior variance and mean respectively. Then,

$$V_{pm}^{\Xi_2} = \Big(\sum_i T_i(t)^T \Sigma_1^{-1} T_i(t) + \text{diagonal}(\omega_r) \Big)^{-1},$$

where ω_r is vector of length r with ω replicated r times, and

$$M_{pm}^{\Xi_2} = V_{pm} \sum_i T_i(t)^T (Y_{it} - \Psi_i \Gamma_2 \zeta_2(t))$$

For B-spline coefficient matrices β_1, β_2 and β of dimensions $r_1 \times K_1$, $r_2 \times K_2$ and $r \times K$ respectively, we can re-write $\zeta_{1i}(t) = (\chi_t^1)^T \beta_{1i}$, $\zeta_{2i}(t) = (\chi_t^2)^T \beta_{2i}$ and $\eta_i(t) = (\chi_t)^T \beta_i$ and $\eta(M(t)) = (\chi_{M(t)})^T \beta$, where χ_t^i is the vector of B-spline bases evaluated at time t with K_i many basis functions having equidistant knots. Similarly, χ_t is the vector of B-spline basis evaluated at time t with K many basis functions having equidistant knots. We can now similarly calculate posterior mean and variances of $vec(\beta_1)$, $vec(\beta)$ and $vec(\beta)$.

4. More Results on Human Mimicry Application

The detailed table of the comparison between TACIFA and two stage methods from Experiment (A) is given below.

Table 1: Prediction MSEs of the first and second time series in Experiment (A) using two-stage methods. The top row indicates the R package used to impute, and the first column indicates the method used to warp. mtsdi could not impute at any of the testing time points in this simulation. The two-stage prediction MSEs are all greater than the TACIFA prediction MSEs (4.25 and 2.21).

	`missForest`	`MICE`	`mtsdi`
Naive DTW	(5.21, 7.2)	(17.44, 14.25)	(5.12, 5.31)
Derivative DTW	(5.21, 7.2)	(19.15, 15.51)	(5.12, 5.31)
Sliding DTW	(5.21, 7.2)	(17.59, 15.63)	(5.12, 5.31)

We present results for two additional experiments. In experiment (B), one individual is instructed to imitate the others smile for the first part of the experiment, but

then the roles are reversed for the second part of the experiment. In experiment (C), the individuals are not doing anything initially, then one individual starts imitating the others smile, and then the participants switch roles later in the interaction.

4.1 Experiment (B)

In this experiment, one individual is instructed to imitate the others smile for 55% of the experiment, and then the roles are reversed for the rest of the experiment. We apply TACIFA and two-stage models to the time courses of six regression scores around the lip and three more predictors on the head position. First we evaluate the loading matrices of the shared and individual factors. Then we assess the ability of TACIFA and two-stage models to identify the known role-reversal at the appropriate time in the experiment, and assess out-of-sample prediction MSEs. Finally, we compute similarity scores using different subsets of predictors.

The two individuals who participated in this experiment intentionally moved very little outside of the movement associated with smiling. As a consequence, we might predict that there should be very few important individual-specific factors. Figure 2 confirms this prediction, as none of the individual-specific loading matrices have high SP values. In contrast, 7 components seem to be important in the shared space loading matrices. Since 6 regression based mouth features were included in this model, we might predict that there would be 6 important factors in the shared space. Accordingly, having 7 important factors in the shared space is not unreasonable.

In addition, TACIFA successfully identifies when the roles of the participants in the experiment switched. In our warping function graphs, the $M(t) = t$ line indicates times when the time points of the shared factor of the two individuals are perfectly aligned. When $M(t) > t$, the first person is leading relative to the second. When $M(t) < t$, the second person is leading relative to the first. Figure 3 illustrates the estimated TACIFA warping function with 95% credible bands, along with the part of the experiment where the direction of mimicry is reversed. For the first part, $M(t) > t$, which correctly implies that the first person is leading, and for the second part of the experiment, $M(t) < t$, which correctly indicates that the second person is leading. Naive DTW and sliding window DTW also show some changes when the roles of the participants in the experiment switched. However, the changes are more prominent for our TACIFA based warping function. Derivative DTW did not detect the direction changes.

The MSEs of out of sample predictions are 0.14 and 0.11 (relative to the estimated variances 0.11 and 0.10), with 88% and 90% frequentist coverage within 95% posterior predictive credible bands, for the first and second individuals, respectively. The prediction MSEs for the two stage methods are all around 0.12 and 0.24 for the two individuals respectively. Detailed results are in Table 2.

Finally, we compute the similarity of the time courses extracted from the experiment. Since the participants were intentionally imitating each others smiles, the similarity between the time courses should be high when the features in the time courses

Table 2: Prediction MSEs of the first and second time series in Experiment (B) using two-stage methods. The top row indicates the R package used to impute, and the first column indicates the method used to warp. mtsdi could not impute at any of the testing time points in this simulation. The two-stage prediction MSEs are all greater than the TACIFA prediction MSEs (0.14 and 0.11).

	`missForest`	`MICE`	`mtsdi`
Naive DTW	(0.11, 0.18)	(0.15, 0.35)	(0.10, 0.20)
Derivative DTW	(0.11, 0.19)	(0.17, 0.33)	(0.10, 0.20)
Sliding DTW	(0.11, 0.18)	(0.17, 0.34)	(0.10, 0.20)

relate to smiles. The similarity should be lower when the features in the time courses do not relate to smiles. To test the second case, X_3 and Y_3 denote the paired time series with only head position data, which are not directly related to smiling. To test the first case, X_6 and Y_6 denote the paired time series with additional 3 smile related features, and X_9 and Y_9 are the complete data set. We get Syn(X_3, Y_3)=0.56. However, as would be expected, the similarity increases dramatically when smile-related features are added to the time series, such that Syn(X_6, Y_6)=0.80 and Syn(X_9, Y_9)=0.85.

4.2 Experiment (C)

In this experiment, the individuals are instructed not to do anything for the first 25% of the experiment, then one individual is instructed to imitate the others smile, and then the roles are reversed for the rest of the experiment. We again apply TACIFA and two-stage models to the time courses of six regression scores around the lip and three more predictors on the head position as the data. Then we evaluate the same set of metrics as in experiment (B).

In Figure 4, the first three components for individual 1 and individual 2 of the individual-specific loading matrices seem to be important, and 7 components seem to be important in the shared space loading matrices. Having three individual specific factors is consistent with having three predictors on head position that are unrelated to the experiment of smile imitation. Again, we might predict that there would be 6 important factors in the shared space. Thus, again , having 7 important factors in the shared space is not unreasonable.

We also find that TACIFA identifies the change points of the experiment more accurately than other methods in Figure 5. The estimated warping function along with credible bands are shown is Figure 5. The interval where the participants start to imitate is marked in this plot as well as the interval where the direction of mimicry is switched. For the initial few time points, the warping function is flat and at around 75% time it intersects the dashed line. There are some changes in naive DTW and

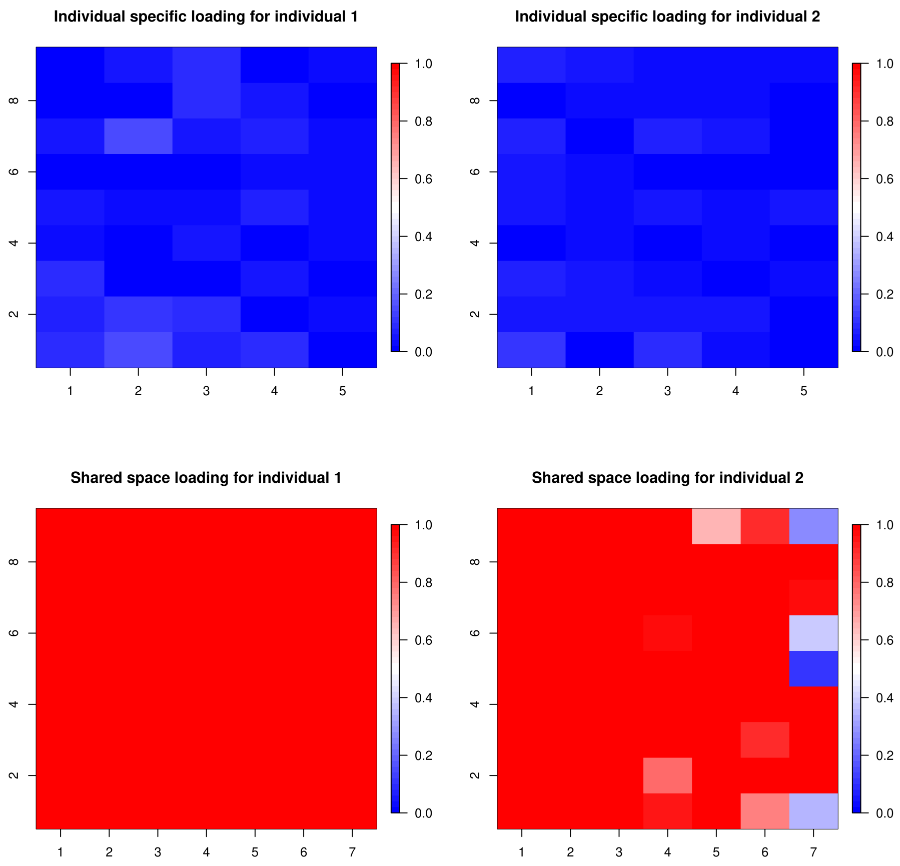

Figure 2: Plot of the summary measure as an evidence of importance of the entries of loading matrices in human mimicry dataset (B). Each column represent each factor. The columns with higher proportion of red correspond to the factors with higher importance.

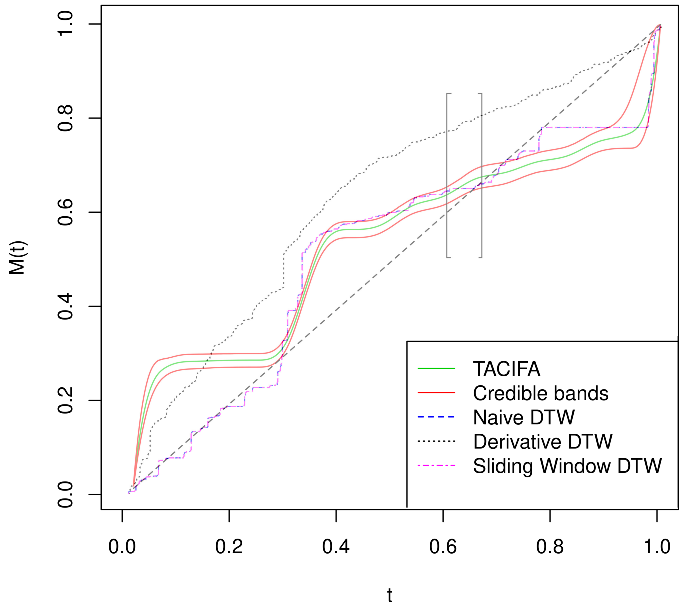

Figure 3: Estimated warping function in human mimicry dataset (B). The green curve is the estimated function along with the 95% pointwise credible bands in red. The portion of the warping function is marked where the direction of imitation is changed in the data.

sliding window DTW curves at these change points on the experiment. However, the changes are more prominent for our TACIFA based warping function in this case also. Once again, derivative DTW did not detect the mimicry direction changes at all.

The MSEs of out of sample predictions are 0.21 and 0.18 (relative to the estimated variances 0.19 and 0.11) with 88% and 90% frequentist coverage within 95% posterior predictive credible bands for the first and second individuals, respectively. The prediction MSEs for the two stage methods are all around 0.17 and 0.35 for the two individuals respectively. Detailed results are in Table 3.

Table 3: Prediction MSEs of the first and second time series in Experiment (C) using two-stage methods. The top row indicates the R package used to impute, and the first column indicates the method used to warp. mtsdi could not impute at any of the testing time points in this simulation. The two-stage prediction MSEs are all greater than the TACIFA prediction MSEs (0.21 and 0.18).

	`missForest`	`MICE`	`mtsdi`
Naive DTW	(0.14, 0.28)	(0.23, 0.36)	(0.14, 0.30)
Derivative DTW	(0.14, 0.28)	(0.30, 0.46)	(0.14, 0.30)
Sliding DTW	(0.14, 0.28)	(0.31, 0.45)	(0.14, 0.30)

Finally, we computed the similarity between the time series of the two individuals. The definitions of X_3, Y_3, X_6, Y_6, X_9 and Y_9 are same as in the previous subsection. We begin by comparing the similarity during the part of the experiment where the individuals were not instructed to imitate each other to the part of the experiment where the individuals did imitate each other. Syn(X_9, Y_9)=0.67 for the non-imitation section and Syn(X_9, Y_9)=0.84 for the imitation section, so the similarity increased, as would be predicted. Next, we tested whether similarity increased when smile-related features are added to the time series, as in the last experiment. We obtain Syn(X_3, Y_3)=0.63, Syn(X_6, Y_6)=0.80 and Syn(X_9, Y_9)=0.85, suggesting similarity does increase as smile-related features are added.

5. More simulations

5.1 Simulation case 1: Direction of mimicry is changed

In this case, we focus on a simulation setting where the direction of mimicry is changed in the middle. The warping function $M(t) = 0.12\sin(2\pi t) + t$. At $t = 0.5$, the direction of mimicry is changed. All the other parameters and functions are generated as in the Simulation case 1 of the manuscript. Then the data X_t and Y_t are generated from $\mathrm{N}(\Psi\zeta_1 + \Lambda\eta(t), 1)$ and $\mathrm{N}(\Psi\zeta_2 + \Lambda\eta(M(t)), 1)$, respectively.

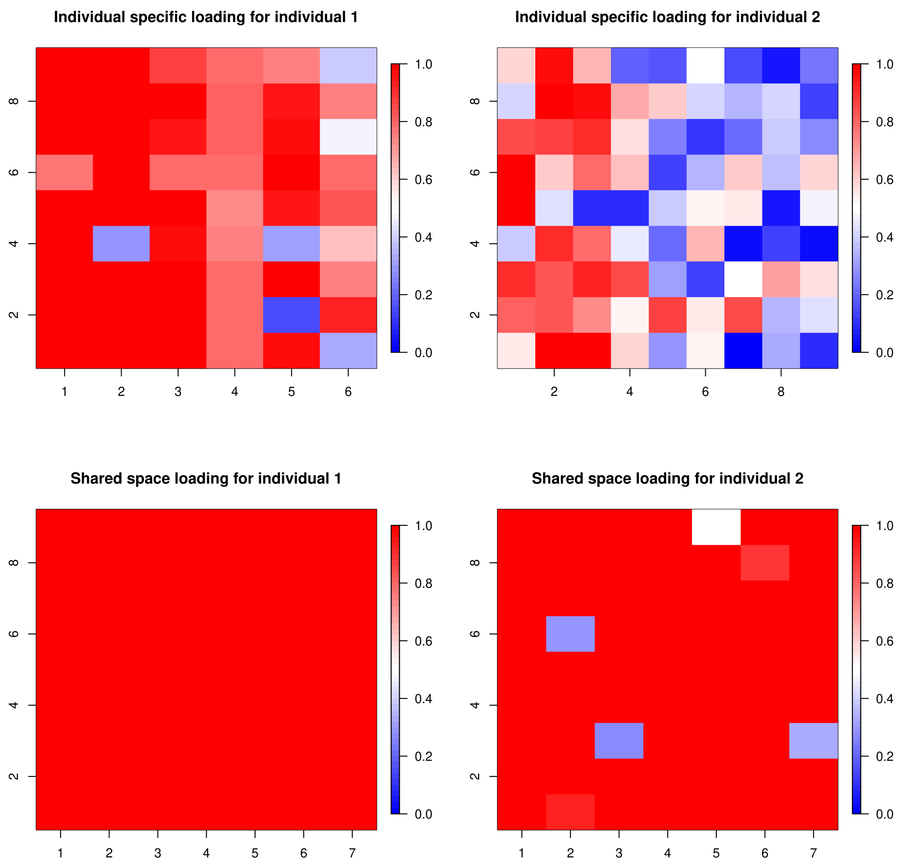

Figure 4: Plot of the summary measure as an evidence of importance of the entries of loading matrices in human mimicry dataset (C). Each column represent each factor. The columns with higher proportion of red correspond to the factors with higher importance.

Table 4: Prediction MSEs of the first and second time series in Simulation 1. using two-stage methods. The top row indicates the R package used to impute, and the first column indicates the warping method. The two-stage prediction MSEs are all greater than the TACIFA prediction MSEs (1.04, 1.10).

	missForest	MICE	mtsdi
Naive DTW	(10.82, 9.81)	(12.18,10.43)	(1.08, 1.17)
Derivative DTW	(10.84, 9.48)	(13.54,11.91)	(1.08, 1.17)
Sliding DTW	(10.88, 8.94)	(10.93,12.15)	(1.08, 1.17)

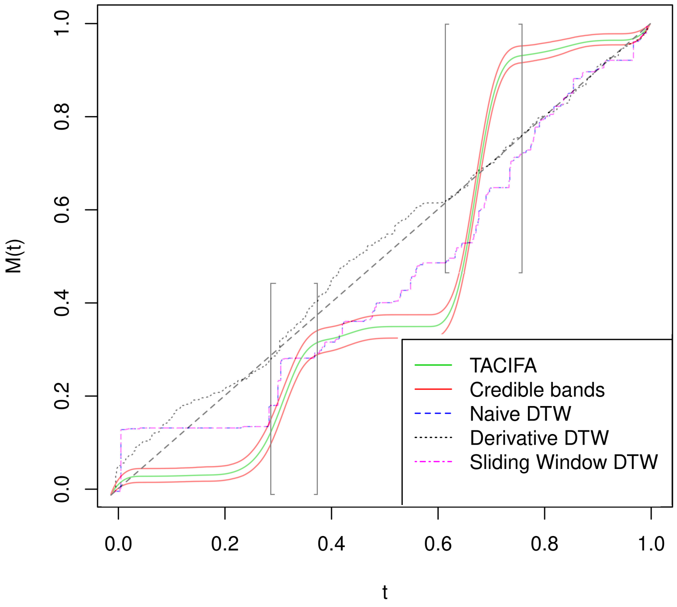

Figure 5: Estimated warping function in human mimicry dataset (C). The green curve is the estimated function along with the 95% pointwise credible bands in red. The portions of the warping function are marked where in the first marked interval the participants start imitating and then in the next marked interval the direction of imitation is changed in the data.

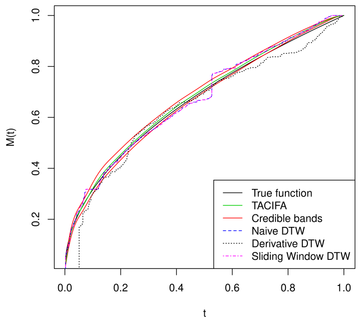

(a) For the case: $\zeta_{1k}(t) = kt$

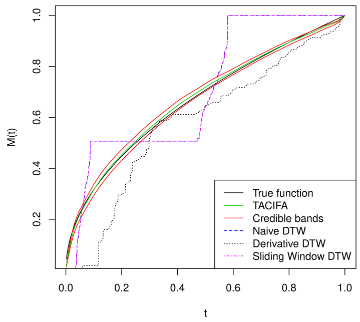

(b) For the case: $\zeta_{1k}(t) = (kt)^2$

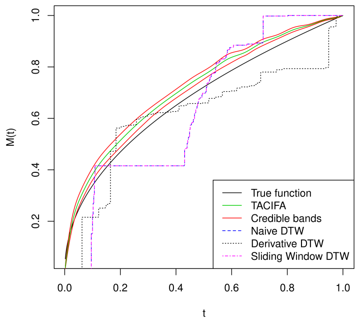

(c) For the case: $\zeta_{1k}(t) = (kt)^3$

Figure 6: Estimated warping functions for different choices of $\zeta_{1k}(t)$. In all of these plots, TACIFA estimated warping is the best. However, as the power in (kt) increases, the estimates are getting worse.

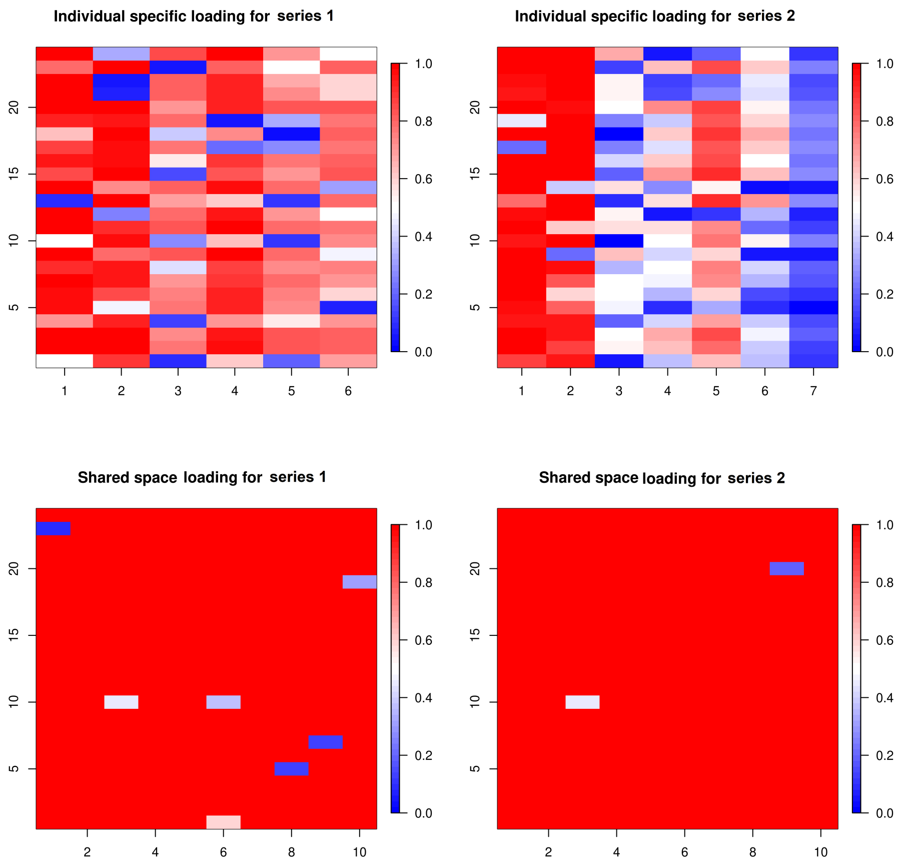

Figure 7: Estimated importance measures SP for loading matrices of shared and individual spaces of Series 1 and 2 in Simulation Case 2. Each column represents a factor. The columns with higher proportion of red correspond to the factors with higher importance.

5.2 Simulation case 2: No-mimicry

In this case, we focus on a simulation setting where there is no mimicry. The data X_t and Y_t are generated from $\mathrm{N}(\Psi\zeta_1+\Lambda\eta(t),1)$ and $\mathrm{N}(\Psi\zeta_2+\Lambda(\eta(t)+\alpha_t),1)$, respectively, where $\alpha_t \sim \mathrm{N}(0,0.1^2)$. All the other parameters and functions are generated as in the Simulation case 1 of the manuscript.

The accuracy of our estimated warping function and accompanying uncertainty quantification are evaluated. The estimated warping function in Figure 8 is for the training set. The estimate by TACIFA is clearly the best among all methods tested. In Table 5, we compare the prediction MSE results of our method with two-stage methods, and show that TACIFA has the best performance.

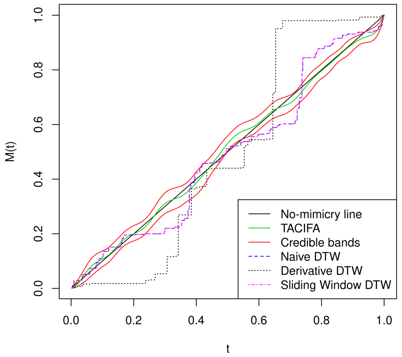

Figure 8: Estimated warping function for simulated data in Simulation Case 1. The black curve is the no-mimicry line $M(t) = t$, the green curve is the estimated function, 95% credible bands are shown in red. Naive DTW and Sliding window DTW curves are indistinguishable. Of all the methods tested, the TACIFA estimated warping function is closest to the true warping function.

Table 5: Prediction MSEs of the first and second time series in Simulation 2. using two-stage methods. The top row indicates the R package used to impute, and the first column indicates the warping method. The two-stage prediction MSEs are all greater than the TACIFA prediction MSEs (1.04, 1.21).

	`missForest`	`MICE`	`mtsdi`
Naive DTW	(10.91, 7.96)	(11.65,11.09)	(1.08, 1.23)
Derivative DTW	(10.86, 7.85)	(10.25,12.93)	(1.08, 1.23)
Sliding DTW	(10.96, 8.11)	(13.10,12.08)	(1.08, 1.23)

References

Subhashis Ghosal and Aad Van der Vaart. *Fundamentals of nonparametric Bayesian inference*, volume 44. Cambridge University Press, 2017.

Bo Ning, Seonghyun Jeong, and Subhashis Ghosal. Bayesian linear regression for multivariate responses under group sparsity. *Bernoulli*, 26:2353–2382, 2020.
